# Structural and Functional Genomics of the Resistance of Cacao to *Phytophthora palmivora*

**DOI:** 10.3390/pathogens10080961

**Published:** 2021-07-30

**Authors:** Jonathan Javier Mucherino Muñoz, Cláusio Antônio Ferreira de Melo, Raner José Santana Silva, Edna Dora Martins Newman Luz, Ronan Xavier Corrêa

**Affiliations:** 1 Department of Biological Science (DBC), Universidade Estadual de Santa Cruz (UESC), Rodovia Jorge Amado km 16, Ilhéus 45662-900, Brazil; jonathanmucherino@gmail.com (J.J.M.M.); clausiomelo@gmail.com (C.A.F.d.M.); ranerbio@yahoo.com.br (R.J.S.S.); 2 Department of Forest Management, Facultad de Ciencias Forestales y Ambientales, Universidad de Los Andes, Mérida 5101, Venezuela; 3 Centro de Pesquisas do Cacau, Comissão Executiva do Plano da Lavoura Cacaueira, Seção de Fitopatologia, Caixa Postal 07, Ilhéus 45600-970, Brazil; ednadora@yahoo.com.br

**Keywords:** QTL, association mapping, *Theobroma cacao*, Black Pod, PAMP, PRR

## Abstract

Black pod disease, caused by *Phytophthora* spp., is one of the main diseases that attack cocoa plantations. This study validated, by association mapping, 29 SSR molecular markers flanking to QTL (Quantitative Trait Loci) associated with *Phytophthora palmivora* Butler (Butler) (PP) resistance, in three local ancient varieties of the Bahia (Comum, Pará, and Maranhão), varieties that have a high potential in the production of gourmet chocolate. Four SSR loci associated with resistance to PP were detected, two on chromosome 8, explaining 7.43% and 3.72% of the Phenotypic Variation (%PV), one on chromosome 2 explaining 2.71%PV and one on chromosome 3 explaining 1.93%PV. A functional domains-based annotation was carried out, in two *Theobroma cacao* (CRIOLLO and MATINA) reference genomes, of 20 QTL regions associated with cocoa resistance to the pathogen. It was identified 164 (genome CRIOLLO) and 160 (genome MATINA) candidate genes, hypothetically involved in the recognition and activation of responses in the interaction with the pathogen. Genomic regions rich in genes with Coiled-coils (CC), nucleotide binding sites (NBS) and Leucine-rich repeat (LRR) domains were identified on chromosomes 1, 3, 6, 8, and 10, likewise, regions rich in Receptor-like Kinase domain (RLK) and Ginkbilobin2 (GNK2) domains were identified in chromosomes 4 and 6.

## 1. Introduction

Cocoa (*Theobroma cacao* L.) is a perennial tree belonging to the Malvaceae Juss. family [1]. Its cultivation represents a livelihood for more than 120 million people in 50 tropical countries, generating more than 12 billion USD per year in revenue, mainly related to the production of chocolate [2,3]. Currently, annual consumption exceeds 4.5 million tons of dried almonds [3,4]. Brazil is the second main cocoa producer in America after Ecuador [3] and the state of Bahia is one of the main producers in the country [5]. In southern Bahia, nearly 70% of cocoa cultivation occurs in Cabruca systems (a type of agroforestry system) [6]. Comum, Pará, and Maranhão were the predominant cocoa varieties in Bahia, until at least 2003, representing approximately 50% of the land cultivated with cocoa in the state [7,8]. These varieties possess unique characteristics, and were naturalized as Bahian cocoa or Bahia local cocoa cultivars, this region is treated as a secondary area of cocoa diversity [8,9]. The Catongo (spontaneous mutant of Comum cocoa) and Maranhão varieties are currently used in the fine cocoa market, they produce less astringent and more flavorful chocolate [10]. However, these varieties have profiles of more susceptibility to different diseases compared to new clonal cultivars produced in response to the outbreak of witches’ broom disease (caused by the fungus *Moniliophthora perniciosa*) in Bahia in 1989 [11].

The Black Pod (BP) disease, caused by species of the genus *Phytophthora* de Bary [12], is one of the world’s most devastating diseases in cocoa cultivation [13] because of its direct effects on reduction of production [14]. Losses varying of 10% up to 100%, depending on of the country, *Phytophthora* species, and climatic zones [15,16,17,18]. The BP in cocoa has an etiology represented by seven species of the genus *Phytophthora* [19]. The most important in the pantropic are *P. palmivora* Butler (Butler) and *P. capsici* Leonian [20]. The mapping of Quantitative Trait Loci (QTL) associated with *Phytophthora* spp. resistance, was performed by several authors [14,21,22,23,24,25,26,27]. Different statistical approaches to identification have been used, ranging from the traditional two-point approach to a multipoint approach using machine-learning algorithms and Hidden Markov Model (HMM) statistical models [21,28,29].

Different molecular markers have been used in the QTLs detection associated with BP resistance, most commonly at present Single Sequence Repeats or Microsatellites (SSR). Brown et al. [22] identified three QTLs associated with BP resistance, using a population of 256 F_1_ individuals from a crossing Pound7 × UF273, the three QTLs were identified using in vivo inoculation of fruits in the field as a phenotyping strategy. More recently, Akaza et al. [14] identified eight QTLs using the PRR (Pod Rot Rate, cumulative % of rotten fruit in relation to the total number of fruits for 3 years) phenotype methodology, and three QTLs using the methodology FOLRES (Foliar disc inoculation test), methodology developed by Nyassé et al. [30]. The authors applied the two methodologies in three segregating populations: (SCA6 × H) × C1 (179 individuals), (P7 × ICS100) × C1 (173 individuals) and (P7 × ICS95) × C1(183 individuals). Barreto et al. [21] identified six QTLs (for resistance to three species *P. palmivora, P. capsici,* and *P. citrophthora*) in a F_1_ (Full-sib) population of 256 individuals derived from the crossing of heterozygous TSH1188 (Trinidad Selected Hybrids) and CCN51(*Colección Castro Naranjal*), all identified by the leaf disc inoculation methodology. These results showed that the range of % Phenotypic Variation (%PV) varies from 1.776 to 27.6%, which evidences the effect of QTLs/genes of major effect and minor effect on the control of the characteristic. On the other hand, LGs and their location varies significantly in all cases studied. Considering the results of these authors, it is very important that in order to be able to use the information obtained by these authors in marker-assisted selection, these QTLs and their flank markers need validation in populations other than those in which they were identified (different genetic background) [31].

In the Plant–Pathogen interaction, the exchange and recognition of molecular signals, represent, since the beginning of the interaction, the possibility of host and pathogen responses to attack or defense strategies. The identification of possible pathogens by the plant’s immune system can occur in two ways: first, the immunity triggered by PAMPs (*Pathogen Associated Molecular Patterns*) usually called PTI (*PAMP-Triggered Immunity*) or MAMPs (*Microbe Associated Molecular Patterns*) [32,33], the second, is the immunity triggered by effectors ETI (*Effector-Triggered Immunity*) [33]. Recognition of PAMPs is done by PRR receptors (*PAMP-Recognition Receptor*) that can be located on the cell membrane or inside the cell, activating a cascade of signal transduction in the host cell [34,35,36,37]. If the pathogen is adapted to bypass PTI, the susceptibility triggered by effectors develops (ETS–*Effector Triggered Susceptibility*), in which the pathogen secretes effector molecules with the function of suppressing the plant’s possible combat responses [38,39]. Some plants have an effector recognition system by means of Resistance Proteins (R). If the plant has R proteins that recognize the effector molecules, it results in an incompatible reaction and the pathogen is unable to develop in the host tissues (resistance), on the contrary, when the plants do not have these recognition proteins, the compatible reaction occurs and the disease develops in the host (susceptibility) [40,41,42]. So far 13 classes of proteins in plants have been classified as PRR and R (CN, CNL, MLO-like, N, NL, RLK, RLK-GNK2, RLP, RPW8-NL, T, TNL, and TNL and Unknows), based on the presence and combination of domains like NBS (Nucleotide Binding Site), LRR (Leucine-Rich Repeat), TIR (Toll/Interleukin-1), CC (Coiled-Coil), STK (Serine-Threonine Kinase), K (Kinase), RLK (Receptor-like Kinase), MLO (Domains like those present in *Mildew resistance Locus O*), RPW8 (Domains like those present in Powdery mildew resistance protein) and GNK2 (Ginkbilobin2) [43,44,45,46,47].

In this study, we aimed to: (1) test the validity of SSR markers, reported as associated with resistance to BP, in samples of local cocoa cultivars from Bahia; and (2) identify *in-silico* candidate resistance genes within these QTLs regions, based on in structural patterns. Here, we find 4 loci associated with resistance in local cocoa varieties, which may facilitate future breeding programs based on selection assisted by molecular markers and we are also reporting 164 and 160 candidate genes, in CRIOLLO and MATINA genomes respectively, which provide a basis for future functional characterization of resistance-related genes to BP in cocoa.

## 2. Materials and Methods

### 2.1. Plant Materials

The biological material used in this study was obtained from the leaves of 36 cocoa trees, located in the farm Novo Horizonte, Uruçuca, Bahia, Brazil (14° 36.907′ S and 39° 15.891′ W). Twelve individuals were selected in each of the three main diversity groups reported by Rhodes [48]. Within these 12 individuals per group of diversity, represented the three ancient local varieties (ALV) (Comum, Pará, and Maranhão) reported by Santos et al. [8] (Table S1) and for each subgroup of individuals from each ALV, phenotypically contrasting individuals were selected for the *P. palmivora*/*T. cacao* interaction, previously phenotyped by Rhodes [48], using the foliar disk methodology proposed by Nyassé et al. [30,49], the method allowed to select individuals with the highest level of susceptibility to the pathogen and individuals with the minimum level of susceptibility (resistant). Additionally, four controls were used: (1) SCA6 as resistant [12,50,51], (2) SIC23 reported as moderately resistant [52], (3) TSH1188 reported as moderately resistant or resistant [50], and (4) Catongo reported as susceptible [51]. The plant material of these controls was collected in the *Banco Ativo de Germoplasma of Centro de Pesquisa do Cacau* (CEPEC/CEPLAC) located in the Km 22 of the highway Itabuna/Ilhéus, Ilhéus, Bahia, Brazil.

### 2.2. DNA Extraction and PCR Amplification

The Cetyl Trimethyl Ammonium Bromide (CTAB) method proposed by Doyle and Doyle [53] was modified, for the extraction of total genomic DNA from leaves of 36 individuals of the ALV and the controls. Proteinase-k was added to the extraction buffer at a final concentration of 400 μg/mL, all centrifugation times were duplicated and performed at a temperature of 4 °C, a further purification of the nucleic acids was carried out with phenol (pH 8.0, equilibrated, ultrapure), chloroform and isoamyl alcohol in a proportion 25:24:1. The final precipitation of the nucleic acids was carried out with isopropanol at a volume of 1:1 with the sample. Finally, three washes were performed, two times with 70% ethanol and once with 95% ethanol and a final resuspension with 100 μL of TE (10 mM Tris-HCl, 1 mM EDTA (pH; 8.0)). The integrity and quantity of extracted DNA was visually quantified in 1.5% agarose gel comparing the samples with a standard lambda phage marker (λ 100 ng). The purified DNA solutions were subsequently diluted with water to a final concentration of 5 ng/μL before use for microsatellite analyses.

### 2.3. SSR Analysis

Thirty-six SSR markers flanking the 20 QTLs associated with resistance to BP reported by Barreto et al. [21], Akaza et al. [14], and Brown et al. [22] with a distance less than 10 cM, in the respective linkage maps, from each QTL were selected. The authors, sequences of the primers and other information of the markers are described in Table S2. The mixture for polymerase chain reaction (PCR) amplification, up to a final volume of 13 µL with water, contained 15 ng template DNA, 0.131 mM forward primer 5′ labeled with the M-13 tail sequence 5′-CACGACGTTGTAAAACGAC-3′, 0.5 mM reverse primer, 0.5 mM M-13 fluorescent DYE (one for each SSR marker, 6-FAM™, NED™, PET^®^, VIC^®^), 0.254 mM dNTP mix, 1.5 mM MgCl_2_, 15.7 nM BSA (bovine serum albumin, supplied in 10 nM Tris-HCl (pH 7.4 at 25 °C), 100 mM KCl, 1 mM EDTA, and 50% (*v/v*) glicerol), 10× PCR buffer (200 mM Tris-HCl (8.4), 500 mM KCl), and 1 U of Taq polymerase (Invitrogen, SP, Brazil). The PCR reactions were performed using a Veriti 96-Well Thermal Cycler (Applied Biosystems); the PCR program included an initial denaturation at 94 °C for 5 min, followed by first 30 cycles of 30 s denaturation at 94 °C, 45 s for annealing at 55 °C, and 45 s extension at 72 °C, then eight cycles of 94 °C denaturation for 30 s, 53 °C annealing for 45 s, and 72 °C extension for 45 s, and a final extension at 72 °C for 10 min. The PCR amplifications quality and approximate size of the amplicons were evaluated on 1.5% agarose gel, using a 100 bp DNA Ladder. Tetraplex systems of 10 µL of final volume were prepared, containing 7.8 µL HI-DI formamine, 0.2 µL GeneScam-500 LIZ^®^ size standard, and 2 µL of mixture contained 0.5 µL of each four PCR products (each labeled with a different DYE, Table S2). The samples were denatured for 5 min at 94 °C, immediately chilled on ice, then capillary electrophoresis was performed on an ABI 3500 Genetic Analyzer (Applied Biosystems) using POP-7™ polymer (Applied Biosystems), 50 cm capillary arrays and default instrument settings. GeneMarker^®^ software (V2.6.3, SoftGenetics) was used to visualize, establish the peaks of filtering (RFU Relative Fluorescent Units) and interpretation, define the genotype of each individual, and generate the compilation of genotype data.

### 2.4. Genome Alignment

In order to obtain the physical coordinates of the different markers, the sequences of the markers were downloaded in FASTA format available in the database GenBank of National Center for Biotechnology Information (NCBI) and aligned, using the BLASTn [54] algorithm, aligning the sequence of the markers with the V2.0 of the CRIOLLO genome (GCF_000208745.1, total size 324.7 Mb, scaffolds N50 = 6.5 Mb, 96.7% anchored, and Unknown sites 5.7%) [55], and with the V1.0 of the Matina 1–6 genome (GCF_000403535.1, total size 346 Mb, contigs N50 = 84.4 Kb, scaffolds N50 = 34.4 Mb, 95.5% anchored and Unknown sites 4.4%) [56]. An expected value (E-value) of 10 (e-10) was used to obtain the alignment with a lower probability of detecting false positives and a maximum number of 20 aligned sequences to keep. Additionally, an alignment was made of the sequences of the primers (Table S2) (in silico PCR) of each marker, published by the different authors and validated with the PROBE database of NCBI, with an expected amplicom size between 40 to 1000 bp, and a single mismatch was considered acceptable.

### 2.5. Analyses of Population Structure

The population structure of the individuals was estimated through the principal components analysis (PCA). It was necessary to adapt the information resulting from the capillary electrophoresis and the GeneMarker^®^ software, in diploid HapMap (Haplotype Map) PHASE format using the IUPAC (International Union of Pure and Applied Chemistry) nomenclature [57]. Each allele per locus was encoded as a SNP (Single nucleotide polymorphisms) allele, the information of the physical coordinates of each locus resulting from the alignments made with the BLASTn algorithm was also included. This genotype data set was transformed to probabilistic numerical values (homozygous major is 1.0, homozygous minor is 0.0, and heterozygous is 0.5). PCA analysis was performed using a covariance matrix, the number of main components was selected based on the ratio of the number of components and the total variability covered with the addition of a new component.

The level of relatedness among the individuals of the population was obtained using the CLADOGRAM plug-in of TASSEL^®^. The distance model between each taxon used by TASSEL^®^ is a modified Euclidean distance, the model treats a homozygote as 100% similar to itself and a heterozygote as only 50% similar to itself (due to the two different alleles are present). Two different approaches were used to analyze the distance matrix: Neighbor-Joining and Unweighted Pair Group Method with Arithmetic mean (UPGMA). The trees generated by the plug-in were visualized using FigTree software.

### 2.6. Association Mapping

The level of correlation between allelic variations and phenotypic variations and the effect of each allele per marker was evaluated on the phenotypic values of each individual using the GLM (General Linear Model) plug-in of TASSEL^®^ [58], a unified matrix with phenotypic, genotypic (transformed to probabilistic values), and structure information was used as input. A test of 1000 permutations was carried out, based on the method proposed by Anderson and Ter Braak [59], which calculates the predicted and residual values of the reduced model, then permutes the residuals and adds them to the predicted values.

### 2.7. Retrieving of the Protein Sequences

Once the physical coordinates of the flanking markers of the QTLs were located, the information contained between each pair of flanking markers were downloaded. This information was downloaded directly using the services of JBrowser [60] to the CRIOLLO genome, and GBrowser [61] to the MATINA genome. For the CRIOLLO genome, a direct download of the proteins predicted in the regions was also made in FASTA format. For the genome MATINA it was necessary to use an *in-house* script in Perl language for the Retrieving of the predicted proteins for each genomic region contained in the GFF3 files, and later transform the protein sequences in FASTA format.

### 2.8. Alignment and Functional Annotation of Protein Sequences Based on Domains

The retrieved FASTA files containing the protein sequences of the genomic regions of the two genomes were analyzed with the InterProScan [62] package compendium, version (V5.50-84.0). The package was requested to compare the sequences with the following databases: TIGRFAM (V15.0), SFLD (V4), SUPERFAMILY (V1.75), PANTHER (V15.0), Gene3D (V4.2.0), Hamap (V2020_05), Coils (V2.2.1), ProSiteProfiles (V2019_11), SMART (V7.1), CDD (V3.18), PRINTS (V42.0), ProSitePatterns (V2019_11), Pfam (V33.1), MobiDBLite (V2.0), and PIRSF (V3.10); the output format was *Tab Separated Values File* (TSV). Additionally, the recovery of the GO terms (TGOC, 2018) associated with each of the proteins was requested (-goterms).

### 2.9. Classification of Genes Associated with Resistance to Pathogens in Plants

The files in tabulated TSV format generated by the InterProScan were used as input for the software RRG_Predictor [63], which makes a search in two steps, the first searches for sequences with one or more domain codes associated with PRR and R genes, and the second step classifies those sequences into one of the 13 resistance gene classes in plants associated with resistance to pathogens, based on the following domain combinations: (1) Proteins with Coiled-coils (*CC*) and nucleotide binding sites (NBS) domains, (2) proteins with CC, NBS, and Leucine-rich repeat (LRR) domains, (3) proteins with Mlo-like resistance proteins, (4) proteins with just NBS domain, (5) proteins with NBS and LRR domains, (6) proteins with Receptor-like Kinase (RLK), (7) proteins with RLK and Ginkbilobin2 (GNK2) domains, (8) proteins with Receptor-like without the Kinase domain, (9) proteins with Resistance to Powdery Mildew 8 (*RPW8*) NBS, and LRR domains, (10) proteins with Toll/interleukin-1 receptor (TIR) domain, (11) proteins with TIR and NBS domains, (12) proteins with TIR, NBS, and LRR domains, and (13) proteins with Leucine-rich repeat (LRR) domains that do not fit any other class (UNKNOWN).

### 2.10. Alignment and Functional Annotation of Complete Protein Sequences

A subset of proteins was generated containing the sequences of the positive hits resulting from the RRG_Predictor analysis for each genome, these proteins sequences were aligned against the “nr” protein database of the NCBI, V04_4_2021. The alignment was made using DIAMOND [64], using the parameters: an expected e-value of 1 × 10^−5^ (e 1e-5), a maximum number of 3 best alignments (-k 3), the results were requested in tabular text format (-f 6), the rest of the parameters were left in default for all analyzes. Word cloud of annotation field for both genomes were produced in the wordclouds.com, ignoring stop terms, connectors, and words not associated with functions.

### 2.11. Functional Annotation in Gene Ontology Language

Using the subset of proteins with positive hits resulting from the analysis with RRG_Predictor, the GO codes associated with each of these proteins, result of the analysis with InterProScan, were recovered (Folder S1_INTERPROSCAN), then we used WEGO [65] to analyze and plot GO annotations for both genomes.

## 3. Results and Discussion

### 3.1. SSR Analysis

For the 1160 loci (29 markers × 40 individuals), only 44 did not amplify (Folder S1_ABI3500), with an average of 1.1 loci per individual without amplification, which generated enough genotypic information for subsequent analyses. The criterion for identifying the quality of the amplification and allelic profile was defined in GeneMarker^®^ software (V2.6.3, SoftGenetics), in which a minimum RFU (Relative Fluorescent Units) cutoff criterion of 150 was used as proposed by Flores and Krohn [66], with well-defined peaks (Figure S1), avoiding flattened peaks of great amplitude, which could generate errors in the identification of the alleles. The standard deviation in the variation of the amplicons attributed to the same allele was evaluated, being 4.5 bp and a difference in length between the alleles of the same locus varying between 10 and 35 bp. From the 29 amplified loci, all were polymorphic, the number of alleles per locus range from two (mTcCIR184, mTcCIR273, and mTcCIR237) up to six alleles per locus (mTcCIR410 and mTcCIR37) (Table 1). The proportion of heterozygous individuals per locus varying from 0 to 0.9 (with an average of 0.43), which is also 0 (mTcCIR273) to 36 (mTcCIR337) (average of 16 individuals) of the 40 individuals heterozygous for the same locus (Table 1). Relatively low values were obtained, for an unstructured free-cross population; a result that contrasts with what was expected due to the high diversity reported by some authors [9,67,68], but being a result homologous to the low diversity reported by Santos et al. [8], about populations of ALV around different municipalities of Bahia state, therefore the present study confirms these findings.

The low diversity observed in the present study is probably associated to the origin of the varieties. All three varieties evaluated are direct descendants of varieties of Amelonado, which also has a low genetic diversity described by different authors [69,70]. Santos et al. [8] also reported a high positive index of fixation, explained by its mating system, which like other traditional cultivars is self-compatible [8,71,72,73].

Historically, the local cocoa varieties of Bahia, come from a limited number of seeds [8] and limited number of genotypes were introduced for more than 200 years. Both factors have increased the levels of inbreeding of these varieties.

### 3.2. Structure and Relatedness

**Figure 1:**
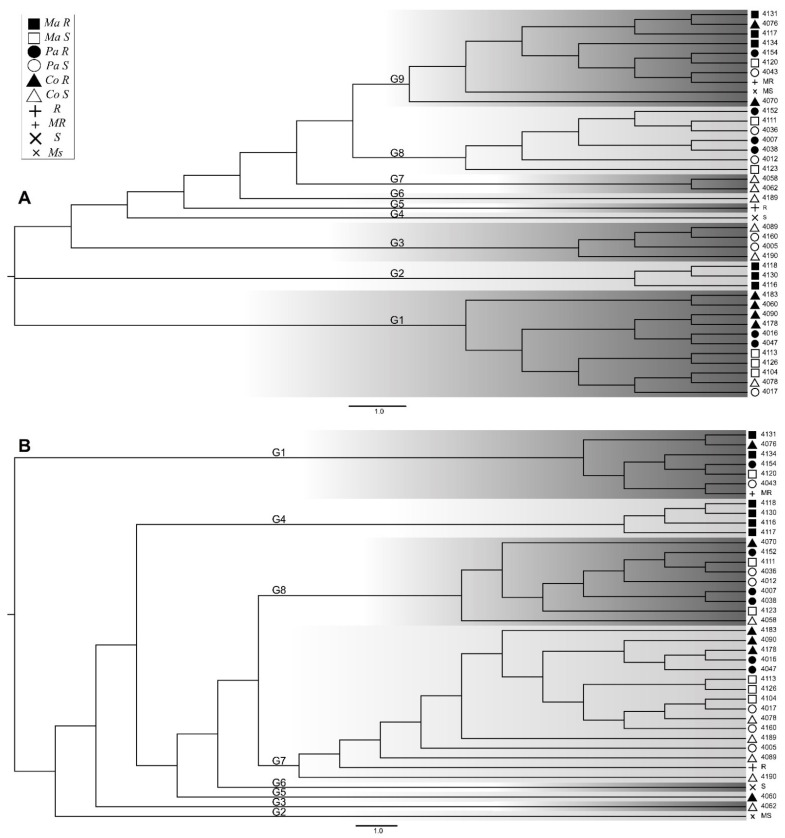
Phylogenetic relationships between Ancient Local Varieties of cocoa (Comum, Pará, and Maranhão). (**A**) Neighbor Joining *Phylogenetic Tree.* (***B***) Unweighted Pair Group Method with Arithmetic Mean *Phylogenetic Tree.* Ma R: Maranhão Resistant, Ma S: Maranhão Susceptible, Pa R: Pará Resistant, Pa S: Pará Susceptible, Co R: Comum Resistant, Co S: Comum Susceptible, R: Resistant, MR: Moderately Resistant, S: Susceptible, Ms: Moderately Susceptible, G1-9: Phylogenetic Groups.

For the NJ analysis (Figure 1A) the formation of nine main groups can be observed, not very different from the eight main groups generated by the UPGMA analysis (Figure 1B), and in general the groups contain almost the same individuals, which shows that genetic recombination between ancestry was greater within the eight or nine main groups formed than it was between these general groups. There were certain similarities between the two phylogenetic analyses, such as the formation of almost pure groups of resistant Maranhão in the two trees G2 NJ and G4 UPGMA. It can be seen that the three individuals that make up the G2 in NJ are present in the G4 of UPGMA, and can be considered as the same group in the methods, appreciating in the UPGMA tree, that the two remaining resistant Maranhão individuals are in an immediate group (G1 UPGMA), highlighting the grouping of all resistant Maranhão individuals into two contiguous groups for the method, showing a clear separation in the method for the contrasting phenotypes in the Maranhão variety, grouping relatively together all the resistant individuals and the susceptible individuals were distributed in the other groups. In the G1 of NJ a differentiated grouping according to the study phenotype can be observed, forming two groups, one with six only resistant individuals (four Comum and two Pará) and the second subgroup only with susceptible individuals with representatives of the three varieties (three Maranhão, one Comum, and one Pará), again with the Maranhão group tending to group differentially, if these subgroups are compared with the UPGMA tree. It can be seen that two resistant Comum individuals (4090 and 4178) and two resistant Pará individuals (4016 and 4047) form an exactly equal subgroup of the G7 in the UPGMA tree, and the other subgroup of only susceptible individuals of the NJ G1 has an almost exact homologous grouping in an internal branch of the G7 in UPGMA. In the NJ tree, the formation of a G3 basal group composed of only susceptible individuals from Comum and Pará can also be observed.

For the Principal Components Analysis (PCA), it was first evaluated what would be the minimum number of components to cover the greatest amount of variability, leaving only the components with the greatest influence on the distribution of the variables. They were reduced to two main components based on the comparison of the amount of total proportion of the variability covered with the inclusion of new components (Figure S2), with the decrease of the components to two, more than half (0.52) of the total proportion of the variability is included.

**Figure 2:**
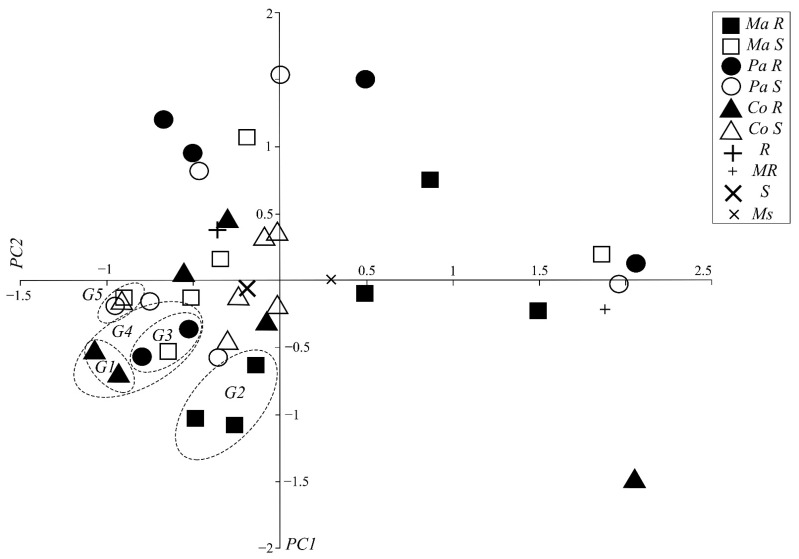
Principal Components Analysis of 36 SSR loci of Ancient Local Varieties of cocoa (Comum, Pará, and Maranhão). PC1: Principal Component 1, PC2: Principal Component 2, Ma R: Maranhão Resistant, Ma S: Maranhão Susceptible, Pa R: Pará Resistant, Pa S: Pará Susceptible, Co R: Comum Resistant, Co S: Comum Susceptible, R: Resistant, MR: Moderately Resistant, S: Susceptible, Ms: Moderately Susceptible.

Once the number of main components was selected, the two-dimensional distribution of the individuals was plotted (Figure 2). It can be seen that there is no clear formation of pure groups, but by comparing the formed subgroups, several homologies with the NJ and UPGMA phylogenetic trees can be seen. The G1 of the PCA analysis contains the same two individuals (4090 and 4178) of the Comum variety and resistant phenotype grouped together in both the NJ (G1) tree and a subgroup of the G7 of the UPGMA tree. The G2 group contains three Maranhão resistant individuals (4130, 4116, and 4118) that correspond exactly to the three individuals of the variety and phenotype grouped in the G2 of NJ and are 3 of the 4 grouped in the G4 of UPGMA. The G3 includes exactly the same two individuals grouped in the Pará resistant subgroup (including noise from a susceptible Maranhão individual) in the G1 NJ and a subgroup of G7 that includes that variety and phenotype in UPGMA. G4 (containing G3 and G1) contains individuals grouped very closely in groups 7 of UPGMA and 1 of NJ. The G5 contains three susceptible individuals (4104, 4078, and 4017) grouped very closely in a sub-branch of the G1 of NJ.

Because the varieties have been planted and mixed for more than 200 years, the crosslinks have resulted in them no longer being genetically distant varieties, despite still possessing very distinctive morphological characteristics [48], such as the shape of the fruit and the taste of the chocolate they produce [8]. Santos et al. [8] observed the differentiation of these varieties can be seen in the separation of individuals from foreign cultivars, such as TSH118 (G2 UPGMA). These authors showed that a more noticeable separation could be appreciated with non-local clones and cultivars than among local varieties, not forming differentiated groups among local varieties. When comparing the results of the three analyses, a marked tendency is observed to the differentiated grouping of the Maranhão variety, specifically the resistant phenotypes, inferring that this group of plants is genetically more distant from the other two varieties. In general, several pure subgroups are denoted in the most basal branches of the trees, indicating the existence of varietal genetic subgroups, mainly for Maranhão and the varieties Comum and Pará being more difficult to differentiate genetically, which can be extrapolated to the difficulty to distinguishing morphologically these two varieties in the field.

### 3.3. Association Mapping

The association of the SSR markers information with the phenotypic data and weighted by the structure, resulted in the identification of four significant loci for resistance to BP at a significant threshold (*p*) ≤ 0.05 by using a GLM approach (Table 2). The threshold (p) was decided based on a QQ (Quantile–Quantile) test (Figure S3), where it was observed that the correlation between the phenotypic values (PP_R_S) and the prediction of the values by the GLM analysis remain overlapped until approximately 0.15 of e-value, therefore a threshold < to 0.05 represents a high reliability.

Two associated markers were detected in the LG8, mTcCIR444 with an e-value (p) of 1.5 × 10^−4^ explaining a 7.43% (R^2^%) of the phenotypic variation and mTcCIR200 with a p = 0.005 explaining a 3.72 %PV, being the markers with the highest level of association to the phenotypic variations. Additionally, one marker was identified in LG2, mTcCIR268 (p = 0.04508) explaining 2.71 %PV and one in LG3, mTcCIR81 with a value of p = 0.0489 explaining 1.92 %PV.

The mTcCIR444 marker showed, a significant value (3.5095 × 10^−6^) for the dominance test (f) compared to the additivity test. This QTL locus was also reported by Akaza et al. [14] with a R^2^% = 13.2 at a cross between (SCA6xH) xC1. The differences observed in the estimated % of the phenotypic variation explained by the locus can be due to two main reasons 1) the genetic difference of the cultivars where the QTL was identified with the genotypes of this study and 2) To the phenotyping methodology that was used, the author used the variable PRR, which was quantified as the accumulated percentage of rotten fruits in relation to the total number of fruits during three years of harvest, in contrast to the foliar disc inoculation method used by Rhodes [48]. Table 3 shows the estimated contribution of locus allele pairs, where favorable alleles contribute significantly to the resistance, the favorable major allele (194 bp) in homozygosis contributes in the decrease of the susceptibility in −1.29 uni (based on the scale proposed by Nyassé et al. [49] (0 maximum resistance and 5 maximum susceptibility)). A second major allele (206 bp) was detected in homozygosis contributing in −1.69 uni and both alleles combined −1.49 uni, confirming the dominance/additivity test.

The second most significant locus, mTcCIR200 also located in LG8, was only reported by Brown et al. [22] with a R^2^% = 7.3, the phenotyping method (PRR) used and the populations also differ (Poun7xUF273) from those used in this study. This locus showed more significant values for the dominance test (0.00748) compared to the additivity test (0.08077). Favorable alleles contribute significantly in resistance: the favorable major allele (293 bp) in homozygosis contributes to a −0.7 uni decrease in susceptibility and the second major allele (303 bp) in homozygosis contributes to a −2.52 uni decrease in susceptibility and when together the major and second major allele to a −1.28 uni. As for the mTcCIR444 marker, the dominance and additivity test revealed consistency. The mtcCIR268 locus located in LG2 was reported by Akaza et al. [14] with a R^2^% = 19.3 and by Barreto et al. [21] with R^2^% = 2.1, with the latter being more consistent with that reported in this study, most likely due to the use of the same phenotyping methodology since this study was conducted in an F1 population of TSH1188xCCN51, both cultivars relatively genetically distant from the local varieties of Bahia. Flament et al. [25] reported a QTL in this same LG explaining 11 %PV in the T60/887xIFC2 cross, IFC2 is a Amelonado of the low Amazon that could be a little more related to the local varieties of Bahia. For the analysis of the estimated contribution of the alleles of this locus, the favorable major allele (367 bp) in homozygosis contributes to a −0.72 uni decrease in susceptibility and the second major allele (350 bp) in homozygosis contributing to a −1.34 uni decrease in susceptibility. The additivity test (0.021) was found to be more reliable than the dominance test (0.91), with an increase in the favorable contribution towards resistance when the alleles were combined.

For the locus mTcCIR81 in the LG3 was also reported by Akaza et al. [14] with a R^2^% = 14.5 and by Barreto et al. [21] with R^2^% = 1.78, again being the reporter value by Barreto et al. [21] more concordant with the finding of this study. Flament et al. [25] also reported a QTL in this LG explaining 9 %PV, but the type of marker used, AFLP, by the authors there is a high level of uncertainty of whether it is located in the same locus reported by the different studies and validated in this work. Risterucci et al. [27] reported a QTL associated with resistance to *P. megakarya*, in the same LG explaining 11.5 %PV and an expected effect of -0.25, the study does not provide information on the primers or selective nucleotides used.

### 3.4. Genomic Localization

It was observed a significant difference in the number of bp of the coordinates when compared between the two genomes. This can be attributed mainly to differences in genotypes sequenced for each genome B97-61/B2 for CRIOLLO and Matina1-6 for MATINA, which have different total sizes. Once the genomic coordinates of each marker were obtained, they were grouped according to their respective QTLs to obtain the flanking coordinates of the QTLs in the two genomes (Table 4), the size of the regions ranged from 198,254 bp to 4,882,174 bp in the CRIOLLO genome (GC) and from 201,785 bp to 7,479,140 bp for the MATINA genome (GM), which may be due to the fact that these regions were obtained based on linkage maps in cM and not on physical maps, adding to that the fact that the cM from each QTL in downstream and upstream direction was very variable in each map and QTL (Max 10 cM downstream and upstream for each QTL), and not all QTLs were reported with two flanking markers but only one, in these cases an average distance of 1.5 MM of base pairs was assumed from the reported marker towards the QTL direction or when the only marker was reported at 0 cM from the QTL, distances of 750,000 bp were assumed in each direction. Two regions with overlaps between the QTLs of different authors were identified, this is because the overlapping QTLs share a flanking marker, the first case was AKAfolICS100CHR1 located completely within the region covered by BARq1BPPcCHR1 in the GC, the second case it was between AKAfolICS100CHR3 and BARq3BPPcCHR3, both positioning practically in the same regions in both genomes.

### 3.5. Selection of Candidate Genes and Functional Annotation

Analysis with InterProScan and RRG_Predictor yielded candidate genes for 11 of the 13 classes (Table 4). A total of 164 candidate genes for the GC and 160 for the GM (Table S3), accumulating the majority in six classes RLK, NL, RLKGNK2, RLP, CNL, and UNKNOWS being the last three those that more hits accumulated in the two genomes. The distribution of the candidate genes in the different genomic regions (Table 4) showed that the two regions richest in candidate genes were Phyto3 (LG10) reported by Brown et al. (2007) and q1-BPPp (LG6) reported by Barreto et al. [21].

Similar results were obtained for some genic classes for the two reference genomes, such as the RLP class, which was also the most represented class, containing hit in 15 of the 20 regions for the GC and 12 regions of the 20 for the GM. However, not all the regions showed such a marked similarity, as for example the CN gene class, in which none of the few hits was located in the same genomic regions of the genomes. These results are probably associated to the different genotypes used for sequencing and the protein prediction algorithms used, ab initio for GM and similarity for GC.

When joining the information of the loci identified as associated to resistance to *Phytophthora palmivora* in this study with the *in-silico* mining of hypothetical genes of resistance (Table 5) it can be observed that almost all the regions (except q2-BPPc LG2) have candidate genes for the GC, being the richest groups PRR/95 (LG2) in the GC reported by Akaza et al. [14] and Phyto2 (LG8) in the GC reported by Brown et al. [22].

An important region is q1-BPPp (LG6), reported by Barreto et al. [21] as associated with resistance to *Phytophthora palmivora* and was the second region richest in candidate genes for the two genomes, accumulating the hits in two main gene classes RLK and RLKGNK2, these class includes RLK (Receptor like Kinase) domains known by Detect specific pathogenic peptides that signal to Pelle-family kinases [74] and play central roles in signaling during pathogen recognition for the subsequent activation of defense mechanisms and developmental control [75], and additionally the RLKGNK2 class contains a GnK2 domain (Ginkbilobin-2), Gnk2 is a protein secreted by *Ginkgo biloba* seeds that exhibits an antifungal activity [76,77]. Gnk2 has a plant-specific cysteine-rich motif DUF26 (domain of unknown function 26, also known as stress-antifungal domain: PF01657) which belongs to cysteine-rich receptor-like kinases (CRKs) [78] not showing any similarity with other known antimicrobial proteins [76,78]. It was recently shown that Gnk2 can also activate actin-dependent cell death [79]. Therefore, *Cast_Gnk2*-like may prevent pathogen growth either by its chemical properties or by inducing HR-related cell death. The hits reported in this genetic class (RLKGNK2), which was the third with the highest number of hits, represents a high potential as candidate genes for *Phytophthora palmivora* resistance in cocoa. In a recent study Santos et al. [80] reported a high differentiation in the expression of *cas_GnK2* in Castanea in trials with inoculation with *Phytophthora cinnamomi*, reporting the high potential that could have the isolation and purification of *Cast_Gnk2*-like protein to the development of an antimicrobial phytopharmaceutical against *P. cinnamomi.*

**Figure 3:**
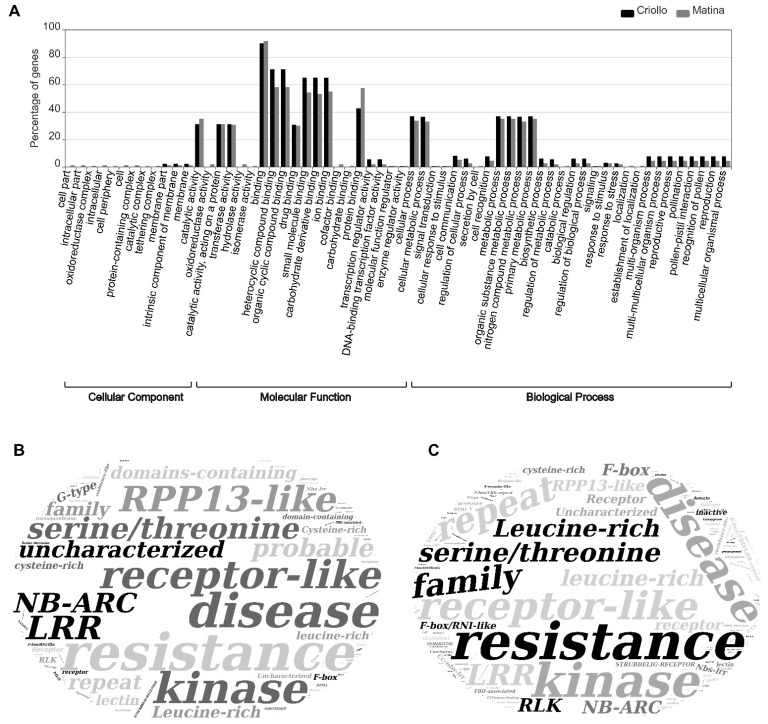
Enrichment Analysis of candidate genes in cocoa involved in the recognition and activation of responses to *Phytophthora palmivora.* (**A**) Gene ontology enrichment of candidate genes for the CRIOLLO and MATINA genomes. (**B**) Alignment-derived function enrichment of candidate genes for the CRIOLLO genome. (**C**) Alignment-derived function enrichment of candidate genes for the MATINA genome.

When graphing the results of the GO terms of the genes identified as candidates (Figure 3A) it was observed that for the Cellular Component level the most common location of the molecules was the cell membrane for both genomes; for the molecular function level, it was observed that the most common function for both genomes was binding; and for the biological process level, metabolic processes were the most common, and less frequently were processes that include multi-organism and multi-cellular organisms. Observing in the three levels, a concordance with the types of targets molecules of the present study that in their great majority are membrane receptors involved in the recognition of pathogens and activation of cascades of response to these pathogens. Observing the result of the Alignment-derived function enrichment analysis of candidate genes for both genomes (Figure 3B,C), it can be seen, in its vast majority structural terms, associated with recognition and resistance to pathogens in plants, expected from this type of molecules, such as: Resistance, Disease, Receptor, Receptor-like, Serine/Threonine, Kinase, Domains-containing, Leucine-rich, LRR, Cysteine-rich, F-box, RLK and NB-ARC among others less frequently. Interestingly, the term RPP13-like was observed with a high frequency for GC and less often for GM. RPP13 is a locus in *Arabidopsis* responsible for the resistance to *Peronospora parasitica* [81,82] organism that taxonomically belongs to the monophyletic group of the Oomycetes, as *Phytophthora palmivora*. The difference in the frequency of the RPP13-like term could be due to the phylogenetic separation and the different levels of resistance to BP between the varieties from which the reference genomes were sequenced [83,84].

## 4. Conclusions

In this study, we present new data of validation of SSR markers in *Theobroma cacao* L. associated with resistance to *Phytophthora palmivora* Butler (Butler) for use in local varieties of Bahia, Brazil, markers never before tested in association with resistance to the pathogen in those varieties with high commercial value for the region and the international chocolate market. Four SSR loci associated with resistance to BP were detected, explaining 1.93 % to 7.43 %PV. The use of these markers in Marker Assisted Selection strategies, in conjunction with early resistance screening techniques, represent a powerful and efficient tool for cocoa breeding programs [85,86]. We also report 164 and 160 candidate genes in the two public genomes available of *T. cacao*, hypothetically involved in the recognition and activation of resistance response to the pathogen, showing that QTLs regions associated with resistance to *P. palmivora* in *T. cacao* are regions rich in genes associated with resistance to pathogens in plants. Genes with RLK and RLK + GNK2 domains, grouped in Chromosomes 4 and 6 and, genes with CNL domains grouped in chromosomes 1, 3, 6, 8, and 10 of cocoa have a great potential to be partially responsible for the recognition and response in the interaction with the pathogen. These genes represent great potential for future functional studies, based on transcriptomics and/or proteomics techniques, to elucidate the molecular defense strategies of cocoa against this pathogen, which has such devastating consequences on the crop.

## Figures and Tables

**Table 1:** Allelic characteristics of SSR loci.

Marker	Major Allele Frequency	Minor Allele Frequency	Allele Number	Number Heterozygous	Proportion Heterozygous
mTcCIR184	0.9375	0.0625	2	5	0.125
mTcCIR118	0.6	0.325	4	30	0.75
mTcCIR273	0.975	0.025	2	0	0
mTcCIR422	0.5625	0.375	5	29	0.725
mTcCIR275	0.7625	0.1625	4	17	0.425
mTcCIR240	0.95	0.05	2	4	0.1
mTcCIR268	0.6625	0.2	5	11	0.275
mTcCIR152	0.55	0.35	4	32	0.8
mTcCIR176	0.7375	0.2375	3	19	0.475
mTcCIR410	0.775	0.1125	6	6	0.15
mTcCIR131	0.6625	0.225	4	21	0.525
mTcCIR81	0.6625	0.3125	3	9	0.225
mTcCIR168	0.4375	0.3375	3	31	0.775
mTcCIR213	0.625	0.175	4	13	0.325
mTcCIR183	0.5125	0.2375	4	24	0.6
mTcCIR95	0.475	0.4	5	31	0.775
mTcCIR237	0.9875	0.0125	2	1	0.025
mTcCIR343	0.5	0.45	4	8	0.2
mTcCIR6	0.5375	0.3	4	3	0.075
mTcCIR136	0.45	0.3625	4	26	0.65
mTcCIR255	0.85	0.1	3	9	0.225
mTcCIR337	0.5	0.425	3	36	0.9
mTcCIR9	0.725	0.125	5	6	0.15
mTcCIR291	0.675	0.1625	4	18	0.45
mTcCIR282	0.5125	0.2125	5	29	0.725
mTcCIR444	0.2375	0.225	5	17	0.425
mTcCIR200	0.5	0.325	4	34	0.85
mTcCIR61	0.7125	0.125	4	5	0.125
mTcCIR37	0.65	0.125	6	8	0.2

**Table 2:** SSR Loci Associated to resistance to Phytophthora palmivora. (LG) Linkage Group, (Perm p) e-value permutations test, (R2 (%)). Percentage of phenotypic variance explained by de locus, (add p) e-value Additivity test, (dom p) e-value Dominance test. (marker df) Marker degrees of freedom, (minorObs) minor allele observations.

Marker Name	LG	Probability of Marker (Marker p)	Perm p	R^2^ (%)	add_p	dom_p	marker_df	minorObs
mTcCIR444	8	0.00015268	0.001	7.4312	2.6313 × 10^−4^	3.5095 × 10^−6^	7	7
mTcCIR200	8	0.005	0.069	3.7166	0.08077	0.00748	5	10
mTcCIR268	2	0.04508	0.521	2.7114	0.02145	0.91435	5	8
mTcCIR81	3	0.0489	0.556	1.9224	0.05314	0.15503	3	9

**Table 3:** Estimated contribution, length of alleles (bp) and number of observations of loci associated with resistance.

Marker	LG	Obs	Allele	Estimate	Allele size (bp)
mTcCIR444	8	5	A	−1.2851	194
mTcCIR444	8	2	C	−6.6662 × 10^−1^	213
mTcCIR444	8	7	G	0.63938	230
mTcCIR444	8	2	T	−1.6195	206
mTcCIR444	8	7	-	−3.9552 × 10^−1^	-
mTcCIR444	8	3	S	0.15355	-
mTcCIR444	8	9	W	−1.4686	-
mTcCIR444	8	5	Y	0	-
mTcCIR200	8	3	A	−7.4174 × 10^−1^	293
mTcCIR200	8	1	C	−2.5248	302
mTcCIR200	8	1	T	1.40417	282
mTcCIR200	8	1	+	−2.4	-
mTcCIR200	8	10	M	−1.2796	-
mTcCIR200	8	24	W	0	-
mTcCIR268	2	21	A	−7.2632 × 10^−1^	367
mTcCIR268	2	2	G	0.10466	371
mTcCIR268	2	4	T	−1.3351	350
mTcCIR268	2	2	+	−1.3891	-
mTcCIR268	2	3	M	−5.9507 × 10^−2^	-
mTcCIR268	2	8	W	0	-
mTcCIR81	3	22	A	−8.7655 × 10^−1^	296
mTcCIR81	3	8	T	−7.0592 × 10^−2^	317
mTcCIR81	3	1	+	0.66546	-
mTcCIR81	3	9	W	0	-

**Table 4:** Number of candidate genes per region and type of domains present. CN: proteins with Coiled-coils (*CC*) and nucleotide binding sites (NBS) domains, CNL: proteins with CC, NBS, and Leucine-rich repeat (LRR) domains, MLO: proteins with Mlo-like resistance proteins, N: proteins with just NBS domain, NL: proteins with NBS and LRR domains, RLK: proteins with Receptor-like Kinase (RLK), RLKGNK2: proteins with RLK and Ginkbilobin2 (GNK2) domains, RLP: proteins with Receptor-like without the Kinase domain, RPW8NL: proteins with Resistance to Powdery Mildew 8 (*RPW8*) NBS and LRR domains, T: proteins with Toll/interleukin-1 receptor (TIR) domain, UNKNOWN: proteins with Leucine-rich repeat (LRR) domains that do not fit any other class, C: CRIOLLO, M: MATINA, *: repeated genes due to overlapping QTL from different authors.

QTL	LG	G	Physical Position	CN	CNL	MLO	N	NL	RLK	RLKGNK2	RLP	RPW8NL	T	UNKNOWN	Total
AKAfolICS100CHR10	10	C	2551520:4052040	-	-	1	-	-	-	-	-	-	-	2	3
		M	16571900:18072415	-	-	-	-	-	-	-	-	-	-	1	1
AKAfolICS100CHR1	1	C	30694434:33468108	-	-	-	-	-	-	-	1	-	-	-	1
		M	28502617:31502617	-	-	-	-	-	-	-	-	-	-	5	5
AKAfolICS100CHR3	3	C	35179055:36293379	-	2	-	2	3	-	-	1	-	-	3	11
		M	29572563:30703073	1	3	-	2	1	-	-	1	-	-	3	11
AKAprrHCHR1	1	C	871438:2417006	-	2	-	-	-	-	-	1	3	-	3	9
		M	867922:2421710	-	2	-	-	-	-	-	1	2	-	3	8
AKAprrHCHR6	6	C	25659781:26148133	-	-	-	-	-	-	-	-	-	-	-	0
		M	16380796:16859704	-	-	-	-	-	-	-	-	-	-	-	0
AKAprrHCHR8	8	C	1022182:2251917	-	-	-	-	-	-	-	3	-	-	1	4
		M	7171923:8400711	-	-	-	-	-	-	-	3	-	-	1	4
AKAprrICS100CHR4	4	C	30388244:30586498	-	-	-	-	-	-	-	-	-	-	-	0
		M	25770441:25972979	-	-	-	-	-	-	-	-	-	-	-	0
AKAprrICS100CHR61	6	C	20000000:21474437	-	1	-	-	1	-	-	6	-	-	1	9
		M	7295362:8795362	-	-	-	-	-	-	-	-	-	-	4	4
AKAprrICS100CHR62	6	C	3494620:5000000	-	-	-	-	-	-	-	1	-	-	2	3
		M	14881286:16381286	-	2	-	-	-	-	-	2	-	-	-	4
AKAprrICS95CHR2	2	C	703843:2174093	-	2	-	-	-	-	-	2	-	-	7	11
		M	731519:1316407	-	1	-	-	2	-	-	-	-	-	7	10
AKAprrICS95CHR4	4	C	19242045:19491718	-	-	-	-	-	-	-	-	-	-	-	0
		M	11207385:11409170	-	-	-	-	-	-	-	-	-	-	-	0
BARq1BPPcCHR1	1	C	30694434:35306336	-	-	-	-	-	1	-	1 *	-	-	-	1
		M	39928772:41428772	-	4	-	-	-	-	-	2	-	-	3	9
BARq1BPPctCHR6	6	C	219626:689235	1	1	-	-	-	-	-	2	-	-	1	5
		M	6827201:7295820	-	2	-	-	-	-	-	2	-	-	1	5
BARq1BPPpCHR6	6	C	23520483:25804709	-	-	-	-	-	10	16	2	-	1	9	38
		M	16717720:19056211	-	-	-	-	-	6	16	2	-	1	9	34
BARq2BPPcCHR2	2	C	8244114:9182779	-	-	-	-	-	-	-	-	-	-	4	4
		M	7336749:8276242	-	-	-	-	-	-	1	-	-	-	4	5
BARq3BPPcCHR3	3	C	35366498:36293379	-	2 *	-	2 *	3 *	-	-	1 *	-	-	3 *	0
		M	29572563:30545307	1 *	3 *	-	2 *	1 *	-	-	1 *	-	-	3 *	0
BARq4BPPcCHR4	4	C	27592136:28579412	-	-	-	-	-	1	-	1	-	-	3	5
		M	21130572:22096856	-	-	-	-	-	1	-	1	-	-	3	5
BROphy1CHR4	4	C	1:1000000	-	-	-	-	-	-	2	1	-	-	-	3
		M	1:1000000	-	-	-	-	-	-	1	1	-	-	-	2
BROphy2CHR8	8	C	2391280:4552442	-	3	2	-	-	-	-	3	-	-	2	10
		M	4898997:7034634	-	3	2	-	-	-	-	3	-	-	2	10
BROphy3CHR10	10	C	16000000:20882174	1	13	-	1	8	-	1	4	-	-	19	47
		M	2511121:9990261	-	9	-	-	4	-	1	4	-	-	25	43
		Total	C	2	24	3	3	12	12	19	28	3	1	57	164
			M	1	26	2	2	7	7	19	22	2	1	71	160

**Table 5:** Cocoa Candidate Genes at loci associated with resistance to Black Pod. LG: Linkage Group, RLP: proteins with Receptor-like without the Kinase domain, CNL: proteins with Coiled-coils (*CC*) nucleotide binding sites (NBS) and Leucine-rich repeat (LRR) domains, MLO: proteins with Mlo-like resistance proteins, NL: proteins with NBS and LRR domains, N: proteins with just NBS domain, RLKGNK2: proteins with Receptor-like Kinase (RLK) and Ginkbilobin2 (GNK2) domains, UN: proteins with LRR domains that do not fit any other class, *: repeated genes due to overlapping QTL from different authors.

		CRIOLLO						MATINA							
	LG	RLP	CNL	MLO	NL	N	UN	RLP	CNL	MLO	NL	RLKGNK2	CN	N	UN
AKAprrHCHR8	8	3	-	-	-	-	1	3	-	-	-	-	-	-	1
BROphy2CHR8	8	3	3	2	-	-	2	3	3	2	-	-	-	-	2
AKAprrICS95CHR2	2	2	2	-	-	-	7	-	1	-	2	-	-	-	7
BARq2BPPcCHR2	2	-	-	-	-	-	4	-	-	-	-	1	-	-	4
AKAfolICS100CHR3	3	1	2	-	3	2	3	1	3	-	1	-	1	2	3
BARq3BPPcCHR3	3	1 *	2 *	-	3 *	2 *	3 *	1 *	3 *	-	1 *	-	1 *	2 *	3 *

## Data Availability

The data presented in this study are available in supplementary material.

## Supplementary Materials

The following are available online at https://www.mdpi.com/article/10.3390/pathogens10080961/s1, Figure S1: Visualization of amplicons in tetraplex, Figure S2: Proportion of Total Variance vs. Number of Principal Components for Principal Components Analysis of 36 SSR loci of Ancient Local Varieties of cocoa (Comum, Pará, and Maranhão), Figure S3: Quantile–Quantile Test of Phenotypic Data, Table S1: Characteristics of plants materials, Table S2: Characteristics of SSR loci., Table S3: Candidate coding genes for recognition, activation, and combat.
