# Supplementary material for: Structural and Functional Genomics of the Resistance of Cacao to Phytophthora palmivora
Source: Pathogens. 2021 Jul 30;10(8):961. doi: 10.3390/pathogens10080961 (PMC8398157; doi:10.3390/pathogens10080961)
Supplement: Supplementary file 1 [file pathogens-10-00961-s001.zip › pathogens-1225547-supplementary/Supplementar/Figure S2.pdf]

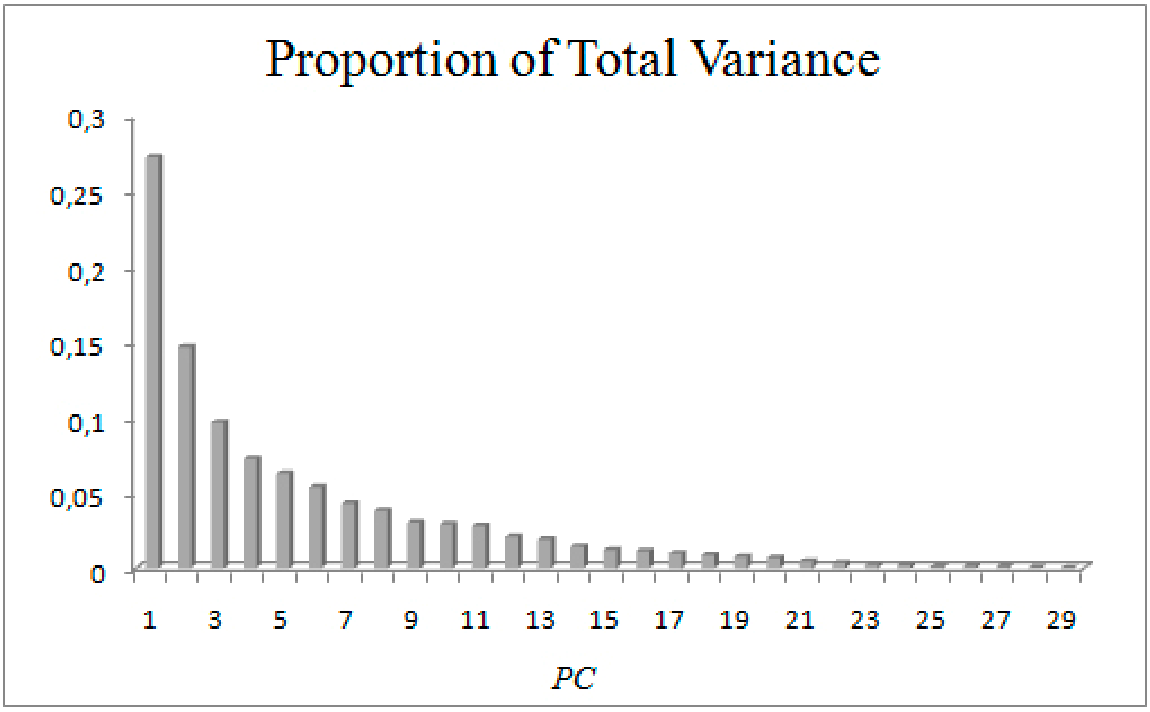

Figure S2. Proportion of Total Variance vs Number of Principal Components for Principal Components Analysis of 36 SSR loci of Ancient Local Varieties of cocoa (Comum, Pará and Maranhão). PC: Principal Components.
