# Supplementary material for: Structural and Functional Genomics of the Resistance of Cacao to Phytophthora palmivora
Source: Pathogens. 2021 Jul 30;10(8):961. doi: 10.3390/pathogens10080961 (PMC8398157; doi:10.3390/pathogens10080961)
Supplement: Supplementary file 1 [file pathogens-10-00961-s001.zip › pathogens-1225547-supplementary/Supplementar/Figure S3.pdf]

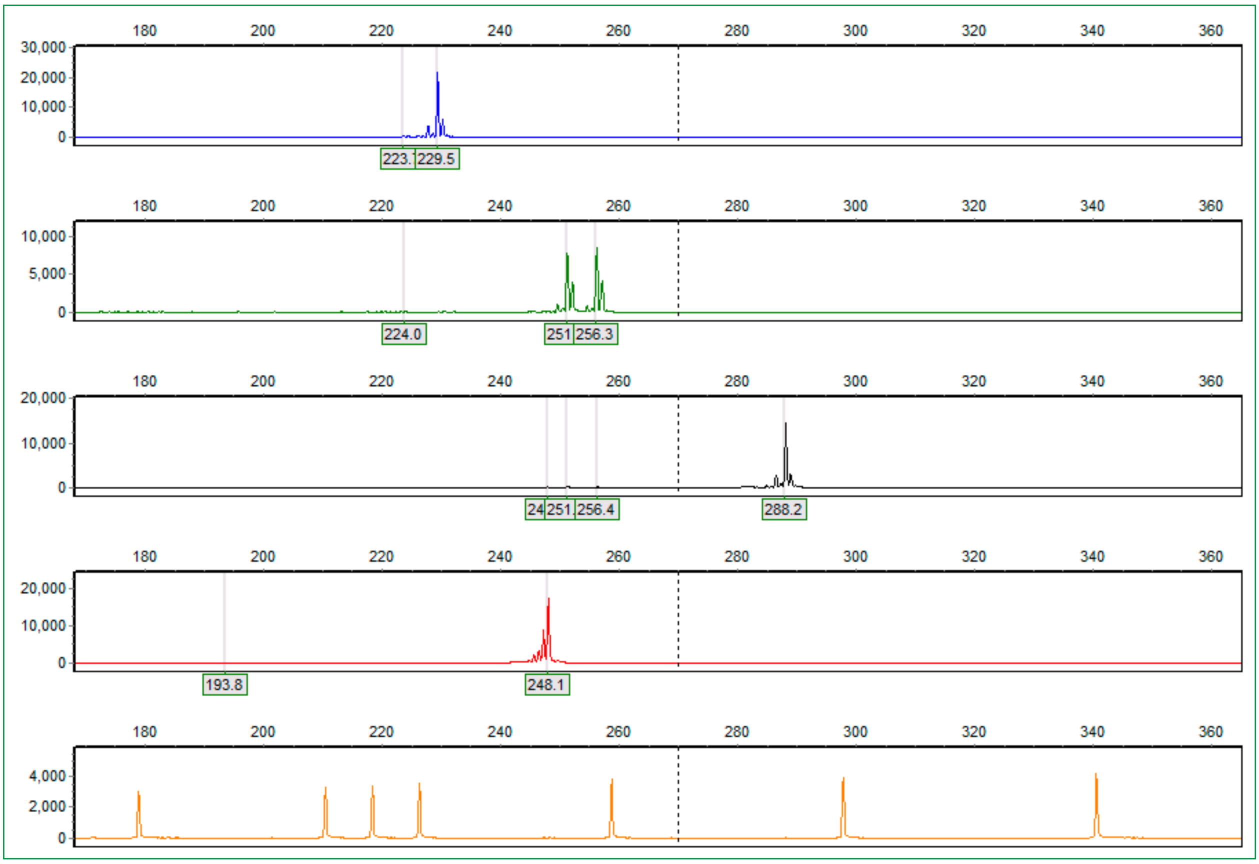

Figure S1. Visualization of amplicons in tetraplex. Sample 4123, markers: mTcCIR273 (PET-Red), mTcCIR275 (6-FAM-Blue), mTcCIR282 (VIC-Green) and mTcCIR291 (NED-Yellow).
