# Supplementary material for: Structural and Functional Genomics of the Resistance of Cacao to Phytophthora palmivora
Source: Pathogens. 2021 Jul 30;10(8):961. doi: 10.3390/pathogens10080961 (PMC8398157; doi:10.3390/pathogens10080961)
Supplement: Supplementary file 1 [file pathogens-10-00961-s001.zip › pathogens-1225547-supplementary/Supplementar/Table S1.pdf]

Table S1. Characteristics of plants materials. (ID) Identifier of individuals, (V) Variety, (FN) Field number, (RL) Raistance level, (PC) Phenotypic class, (PC(S)) Phenotypic class (Simplified).

ID	V	FN	RL	PC	PC(S)
MA_R_4131	Maranhão	4131	1.325	a1	R
MA_R_4118	Maranhão	4118	1.475	a1	R
MA_R_4130	Maranhão	4130	1.775	a1	R
MA_R_4116	Maranhão	4116	1.8	a1	R
MA_R_4117	Maranhão	4117	1.8	a1	R
MA_R_4134	Maranhão	4134	1.8	a1	R
CO_R_4070	Comum	4070	1.575	a1	R
CO_R_4076	Comum	4076	1.4	a1	R
CO_R_4183	Comum	4183	1.55	a1	R
CO_R_4060	Comum	4060	1.625	a1	R
CO_R_4090	Comum	4090	1.825	a1	R
CO_R_4178	Comum	4178	1.925	a1	R
PA_R_4016	Pará	4016	1.725	a1	R
PA_R_4152	Pará	4152	1.85	a1	R
PA_R_4047	Pará	4047	1.95	a1	R
PA_R_4154	Pará	4154	1.95	a1	R
PA_R_4007	Pará	4007	2	a2	R
PA_R_4038	Pará	4038	2	a2	R
MA_S_4113	Maranhão	4113	3.55	a4	S
MA_S_4123	Maranhão	4123	3.55	a4	S
MA_S_4126	Maranhão	4126	3.2	a3	S
MA_S_4111	Maranhão	4111	3.075	a3	S
MA_S_4104	Maranhão	4104	3.05	a3	S
MA_S_4120	Maranhão	4120	3.025	a3	S
CO_S_4058	Comum	4058	4.45	a5	S
CO_S_4189	Comum	4189	4.3	a5	S
CO_S_4078	Comum	4078	3.725	a4	S
CO_S_4089	Comum	4089	3.525	a4	S
CO_S_4062	Comum	4062	3.45	a4	S
CO_S_4190	Comum	4190	3.4	a4	S
PA_S_4012	Pará	4012	3.625	a4	S
PA_S_4043	Pará	4043	3.4	a4	S
PA_S_4036	Pará	4036	3.225	a3	S
PA_S_4160	Pará	4160	3.1	a3	S
PA_S_4017	Pará	4017	3.025	a3	S
PA_S_4005	Pará	4005	2.975	a3	S
S_Cat	Catongo	-	2.969	a3	S
MS_TSH	TSH1188	-	2.824	a3	MS
R_SCA	SCA6	-	1.464	a1	R
MR_SIC	SIC23	-	2.6	a2	MR
