# Supplementary material for: Structural and Functional Genomics of the Resistance of Cacao to Phytophthora palmivora
Source: Pathogens. 2021 Jul 30;10(8):961. doi: 10.3390/pathogens10080961 (PMC8398157; doi:10.3390/pathogens10080961)
Supplement: Supplementary file 1 [file pathogens-10-00961-s001.zip › pathogens-1225547-supplementary/Supplementar/Table S2.pdf]

**Table S2.** Characteristics of SSR loci. (LG) Linkage group, (QTL) quantitative trait loci, (RS) repeated sequence, (DYE) fluorescents used in genotyping, (*) Loci that did not amplify.

| Marker Name | Marker Reference | LG | QTL | QTL Reference | Forward Primer (5' → 3') | Reverse Primer (5' → 3') | RS | DYE |
|---|---|---|---|---|---|---|---|---|
| mTcCIR6 | LANAUD, C. *et al*,1999 | 6 | PRR/100/6.1<br>q1.BP-Pct | Akaza, M. *et al.* 2016<br>Barreto, M. et al 2018 | TAAAGCAAAGCAATCTAACATA | TTCCCTCTAAACTACCCTAAAT | (TG)7(GA)13 | PET |
| mTcCIR9 | LANAUD, C. *et al*,1999 | 6 | PRR/H/6<br>q1.BP-Pp | Akaza, M. *et al.* 2016<br>Barreto, M. et al 2018 | ACATTTATACCCCAACCA | ACCATGCTTCCTCCTTCA | (CT)8N15(CT)5N9(TC)10 | 6-FAM |
| mTcCIR37 | USDA | 10 | Phytoph3 | Brown, J. *et al.* 2007 | CTGGGTGCTGATAGATAAT | AATACCCTCCACACAAAT | (GT)15 | VIC |
| mTcCIR61 | CIRAD/USDA | 10 | Phytoph3 | Brown, J. *et al.* 2007 | GCAGTCTGAAACAAGATAA | TGACTATAATATAAGGCGAA | (CA)18 | NED |
| mTcCIR77* | Pugh T. *et al* 2004 | 10 | FOL/100/10 | Akaza, M. *et al.* 2016 | GTTCTCCCCCACTCTCT | AATAAATAAATAAACAATACG | (TC)9 | - |
| mTcCIR81 | Pugh T. *et al* 2004 | 3 | q3.BP-Pc<br>FOL/100/3 | Barreto, M. et al 2018<br>Akaza, M. et al. 2016 | ACAATCTGTCCATTATTCTG | TGAAACTCCCATACTACTGA | (CT)15 | PET |
| mTcCIR95 | Pugh T. *et al* 2004 | 4 | q4.BP-Pc<br>PRR/100/4 | Barreto, M. et al 2018<br>Akaza, M. et al. 2016 | CATCGTCTTCCTCTCATC | CTCCTTCCCTTTCTCTC | (TC)4 CC | 6-FAM |
| mTcCIR118 | Pugh T. *et al* 2004 | 1 | PRR/H/1 | Akaza, M. *et al.* 2016 | TCTGCCTGAAAATGTCTC | TGGGGCACTAACTTTTG | (GA)10 | VIC |
| mTcCIR131 | Pugh T. *et al* 2004 | 3 | q3.BP-Pc | Barreto, M. *et al 2018* | GATCATCGGTAAAGTAAAAT | TGAGTAAGAAAAAGTAGAAAA | (GA)9 C (GA)4 | NED |
| mTcCIR136 | Pugh T. *et al* 2004 | 6 | PRR/100/6.1<br>q1.BP-Pct | Akaza, M. *et al.* 2016<br>Barreto, M. et al 2018 | GAGGAGGTGAGAGCCA | GGTTTGTATTTTTGATTGAG | (GA)7 GC (GA)7 | PET |
| mTcCIR152 | CIRAD Data Base | 2 | q2.BP-Pc | Barreto, M. *et al 2018* | CAGTAGTCAAAACATCAAA | GTAATCCAAATAATAAGCAT | (TC)9 CC (TC)15 | 6-FAM |
| mTcCIR168 | Pugh T. *et al* 2004 | 4 | Phytoph1 | Brown, J. *et al.* 2007 | GGTAGTATTGAGGTGCGTAT | GTGAATGAATGGATGTGAAA | (TC)9 | VIC |
| mTcCIR176 | Pugh T. *et al* 2004 | 2 | q2.BP-Pc | Barreto, M. *et al 2018* | TCACCAATTCTCTGCTC | AATGAAATTACCTCCTTAC | (TG)16 | NED |
| mTcCIR183 | Pugh T. *et al* 2004 | 4 | PRR/95/4 | Akaza, M. *et al.* 2016 | GTTATCTTAGTTTCTAGCCAC | GTAGTCTTACACCTTGATTG | (AC)9 | 6-FAM |
| mTcCIR184 | Pugh T. *et al* 2004 | 1 | PRR/H/1 | Akaza, M. *et al.* 2016 | GGTTTTCTAGCTCCTCC | AGGAAAGAATGACTCATACTA | (CA)8 (CT)13 | PET |
| mTcCIR200 | Pugh T. *et al* 2004 | 8 | Phytoph2 | Brown, J. *et al.* 2007 | CTTAAAATAAGCCCAAATAC | GCCAATTTCTGACCCA | (TG)8 | VIC |
| mTcCIR202* | Pugh T. *et al* 2004 | 3 | q3.BP-Pc | Barreto, M. *et al 2018* | CCTGAGTCAAAGTGTCCT | TCTCTCATAGCTCAAGCA | (TG)7 (GA)9 | - |
| mTcCIR208* | Pugh T. *et al* 2004 | 6 | PRR/100/6.2 | Akaza, M. *et al.* 2016 | GCAAGCCCCTAAAACT | AAAAAGCAAAAGAAGAAGA | (CT)10-25pb-(AC)11 | - |
| mTcCIR213 | Pugh T. *et al* 2004 | 4 | PRR/95/4 | Akaza, M. *et al.* 2016 | GATCTCGCAAAACTAACA | TAAGTAAAATGAAGGTGTGA | (CT)26 | NED |

| | | | | | | | | |
|---|---|---|---|---|---|---|---|---|
| mTcCIR225* | Pugh T. *et al* 2004 | 8 | Phytoph2 | Brown, J. *et al.* 2007 | AAGACAAAGGGAAGAAGA | AGGGGAAGAGCAAATC | (TC)10 | - |
| mTcCIR237 | Pugh T. *et al* 2004 | 4 | q4.BP-Pc | Barreto, M. *et al 2018* | GAAGACAAGGATGGAGACT | GCAAAGAGAGCAGGAGA | (TC)10 | PET |
| mTcCIR240 | Pugh T. *et al* 2004 | 2 | PRR/95/2 | Akaza, M. *et al.* 2016 | AGTGATTTATGGGACTTT | CATACCTACTACTGCTCTCT | (CT)22 | 6-FAM |
| mTcCIR241* | Pugh T. *et al* 2004 | 4 | PRR/100/4 | Akaza, M. *et al.* 2016 | ACGAGTGAGAGAGTGAAGTT | CAGTTGGAGGGCATTT | (CT)23 | - |
| mTcCIR252* | Pugh T. *et al* 2004 | 2 | PRR/95/2 | Akaza, M. *et al.* 2016 | AATGTGTGCTTTGTTTCTA | TTCAAGGGCGTAACTC | (AC)10 | - |
| mTcCIR255 | Pugh T. *et al* 2004 | 6 | q1.BP-Pp | Barreto, M. *et al 2018* | TTTACCTCCACCATCTT | TGGCACTTATCTATTACTGT | (AC)11 | VIC |
| mTcCIR268 | Pugh T. *et al* 2004 | 2 | q2.BP-Pc<br>PRR/95/2 | Barreto, M. *et al 2018*<br>*Akaza, M. et al. 2016* | CAGTGAAGAGGCAAGAGA | TGTAATCCAAATAATAAGCAT | (GA)17 GG (GA)9 | NED |
| mTcCIR273 | Pugh T. *et al* 2004 | 1 | FOL/100/1<br>q1.BP-Pc | Akaza, M. et al. 2016<br>Barreto, M. et al 2018 | ACGGCATTAGAGAGAGA | AGAATGATCGCAGAGAG | (CT)4 AC (CT)13 TT | PET |
| mTcCIR275 | Pugh T. *et al* 2004 | 1 | q1.BP-Pc | Barreto, M. *et al 2018* | GGTTTGGTTTTGGTAAGAC | TAAGAGAGAGTGATGCTGACA | (CT)11 | 6-FAM |
| mTcCIR276* | Pugh T. *et al* 2004 | 6 | PRR/100/6.1 | Akaza, M. *et al.* 2016 | GTCCTATCTGCCTCACT | TCCTGCTTTTTAATACAT | (GA)14 | - |
| mTcCIR282 | Pugh T. *et al* 2004 | 8 | PRR/H/8 | Akaza, M. *et al.* 2016 | AGCAAAGGCAATAATAATG | TGGTGAGGGGAGAGAA | (GA)2 GG (GA)6 | VIC |
| mTcCIR291 | Pugh T. *et al* 2004 | 6 | PRR/H/6<br>PRR/100/6.2 | Akaza, M. *et al.* 2016<br>Akaza, M. et al. 2016 | AGTCCCATAGGTTCCAAT | CGAGGTTATCCCCAAA | (CT)12 | NED |
| mTcCIR337 | Fouet, O. *et al. 2011* | 6 | PRR/H/6 | Akaza, M. *et al.* 2016 | ACGAAGCCGTAACTTGG | TGCAGGACTCTCTGTCACT | (GGA)5 | PET |
| mTcCIR343 | Fouet, O. *et al. 2011* | 4 | PRR/100/4 | Akaza, M. *et al.* 2016 | GCTTTGCCCTTTCTTCTCT | AGCACTGAACCGAGCAA | (AT)10 | 6-FAM |
| mTcCIR410 | Fouet, O. *et al. 2011* | 3 | FOL/100/3 | Akaza, M. *et al.* 2016 | TTTTGCCTCCCTTGTTCT | TGAATCGTTGAGCGAAAG | (TA)10 | VIC |
| mTcCIR422 | Fouet, O. *et al. 2011* | 1 | FOL/100/1 | Akaza, M. *et al.* 2016 | ACATCCTTTTCTCTGCCTTT | CCCTTCCCATCCCTCT | (GA)18 | NED |
| mTcCIR444 | Fouet, O. *et al. 2011* | 8 | PRR/H/8 | Akaza, M. *et al.* 2016 | TGAACCGGATTGTTGGA | GGGACTTAATCTGGACATGC | (TC)15 | PET |
