# Supplementary material for: Structural and Functional Genomics of the Resistance of Cacao to Phytophthora palmivora
Source: Pathogens. 2021 Jul 30;10(8):961. doi: 10.3390/pathogens10080961 (PMC8398157; doi:10.3390/pathogens10080961)
Supplement: Supplementary file 1 [file pathogens-10-00961-s001.zip › pathogens-1225547-supplementary/Supplementar/Table S3.pdf]

Table S3. Candidate coding genes for recognition, activation and combat. Genome: Reference genome, QTL: Quantitative Trait Loci, GC: Gene Class, Protein ID: Identifier of the protein produced by the gene in its reference genome; *: Repeated genes due to overlapping QTL from different authors.

Genome	QTL	GC	Protein ID
CRIOLLO	AKAfolICS100chr1	RLP	Tc01v2_g027470.1
CRIOLLO	AKAfolICS100chr10	MLO	Tc10v2_g005800.1
CRIOLLO	AKAfolICS100chr10	UNKNOWN	Tc10v2_g004170.1
CRIOLLO	AKAfolICS100chr10	UNKNOWN	Tc10v2_g004280.1
CRIOLLO	AKAfolICS100chr3	CNL	Tc03v2_g026110.1
CRIOLLO	AKAfolICS100chr3	CNL	Tc03v2_g026450.1
CRIOLLO	AKAfolICS100chr3	N	Tc03v2_g026140.1
CRIOLLO	AKAfolICS100chr3	N	Tc03v2_g026480.1
CRIOLLO	AKAfolICS100chr3	NL	Tc03v2_g026160.1
CRIOLLO	AKAfolICS100chr3	NL	Tc03v2_g026170.1
CRIOLLO	AKAfolICS100chr3	NL	Tc03v2_g026190.1
CRIOLLO	AKAfolICS100chr3	RLP	Tc03v2_g027150.1
CRIOLLO	AKAfolICS100chr3	UNKNOWN	Tc03v2_g026130.1
CRIOLLO	AKAfolICS100chr3	UNKNOWN	Tc03v2_g026470.1
CRIOLLO	AKAfolICS100chr3	UNKNOWN	Tc03v2_g026890.1
CRIOLLO	AKAprrHchr1	CNL	Tc01v2_g002300.1
CRIOLLO	AKAprrHchr1	CNL	Tc01v2_g002490.1
CRIOLLO	AKAprrHchr1	RLP	Tc01v2_g001880.1
CRIOLLO	AKAprrHchr1	RPW8NL	Tc01v2_g004360.1
CRIOLLO	AKAprrHchr1	RPW8NL	Tc01v2_g004380.1
CRIOLLO	AKAprrHchr1	RPW8NL	Tc01v2_g004390.1
CRIOLLO	AKAprrHchr1	UNKNOWN	Tc01v2_g002080.1
CRIOLLO	AKAprrHchr1	UNKNOWN	Tc01v2_g002100.1
CRIOLLO	AKAprrHchr1	UNKNOWN	Tc01v2_g003280.1
CRIOLLO	AKAprrHchr8	RLP	Tc08v2_g002170.1
CRIOLLO	AKAprrHchr8	RLP	Tc08v2_g003150.1
CRIOLLO	AKAprrHchr8	RLP	Tc08v2_g003920.1
CRIOLLO	AKAprrHchr8	UNKNOWN	Tc08v2_g003050.1
CRIOLLO	AKAprrICS100chr61	CNL	Tc06v2_g010180.1
CRIOLLO	AKAprrICS100chr61	NL	Tc06v2_g010200.1
CRIOLLO	AKAprrICS100chr61	RLP	Tc06v2_g010130.1
CRIOLLO	AKAprrICS100chr61	RLP	Tc06v2_g010780.1
CRIOLLO	AKAprrICS100chr61	RLP	Tc06v2_g011020.1
CRIOLLO	AKAprrICS100chr61	RLP	Tc06v2_g011030.1
CRIOLLO	AKAprrICS100chr61	RLP	Tc06v2_g011050.1
CRIOLLO	AKAprrICS100chr61	RLP	Tc06v2_g011070.1
CRIOLLO	AKAprrICS100chr61	UNKNOWN	Tc06v2_g011450.1
CRIOLLO	AKAprrICS100chr62	RLP	Tc06v2_g003670.1
CRIOLLO	AKAprrICS100chr62	UNKNOWN	Tc06v2_g003530.1
CRIOLLO	AKAprrICS100chr62	UNKNOWN	Tc06v2_g003690.1

CRIOLLO	AKAprrICS95chr2	CNL	Tc02v2_g001110.1
CRIOLLO	AKAprrICS95chr2	CNL	Tc02v2_g001200.1
CRIOLLO	AKAprrICS95chr2	RLP	Tc02v2_g001540.1
CRIOLLO	AKAprrICS95chr2	RLP	Tc02v2_g002630.1
CRIOLLO	AKAprrICS95chr2	UNKNOWN	Tc02v2_g001020.1
CRIOLLO	AKAprrICS95chr2	UNKNOWN	Tc02v2_g001100.1
CRIOLLO	AKAprrICS95chr2	UNKNOWN	Tc02v2_g001120.1
CRIOLLO	AKAprrICS95chr2	UNKNOWN	Tc02v2_g001130.1
CRIOLLO	AKAprrICS95chr2	UNKNOWN	Tc02v2_g001170.1
CRIOLLO	AKAprrICS95chr2	UNKNOWN	Tc02v2_g001190.1
CRIOLLO	AKAprrICS95chr2	UNKNOWN	Tc02v2_g002950.1
CRIOLLO	BARq1BPPcchr1	RLK	Tc01v2_g029920.1
CRIOLLO	BARq1BPPcchr1	RLP	Tc01v2_g027470.1*
CRIOLLO	BARq1BPPctchr6	CN	Tc06v2_g000460.1
CRIOLLO	BARq1BPPctchr6	CNL	Tc06v2_g000490.1
CRIOLLO	BARq1BPPctchr6	RLP	Tc06v2_g000350.1
CRIOLLO	BARq1BPPctchr6	RLP	Tc06v2_g000550.1
CRIOLLO	BARq1BPPctchr6	UNKNOWN	Tc06v2_g000470.1
CRIOLLO	BARq1BpPpchr6	RLK	Tc06v2_g016800.1
CRIOLLO	BARq1BpPpchr6	RLK	Tc06v2_g016810.1
CRIOLLO	BARq1BpPpchr6	RLK	Tc06v2_g016840.1
CRIOLLO	BARq1BpPpchr6	RLK	Tc06v2_g016860.1
CRIOLLO	BARq1BpPpchr6	RLK	Tc06v2_g016870.1
CRIOLLO	BARq1BpPpchr6	RLK	Tc06v2_g016880.1
CRIOLLO	BARq1BpPpchr6	RLK	Tc06v2_g016900.1
CRIOLLO	BARq1BpPpchr6	RLK	Tc06v2_g016910.1
CRIOLLO	BARq1BpPpchr6	RLK	Tc06v2_g016920.1
CRIOLLO	BARq1BpPpchr6	RLK	Tc06v2_g016930.1
CRIOLLO	BARq1BpPpchr6	RLKGNK2	Tc06v2_g017250.1
CRIOLLO	BARq1BpPpchr6	RLKGNK2	Tc06v2_g017290.1
CRIOLLO	BARq1BpPpchr6	RLKGNK2	Tc06v2_g017300.1
CRIOLLO	BARq1BpPpchr6	RLKGNK2	Tc06v2_g017320.1
CRIOLLO	BARq1BpPpchr6	RLKGNK2	Tc06v2_g017330.1
CRIOLLO	BARq1BpPpchr6	RLKGNK2	Tc06v2_g017340.1
CRIOLLO	BARq1BpPpchr6	RLKGNK2	Tc06v2_g017350.1
CRIOLLO	BARq1BpPpchr6	RLKGNK2	Tc06v2_g017360.1
CRIOLLO	BARq1BpPpchr6	RLKGNK2	Tc06v2_g017370.1
CRIOLLO	BARq1BpPpchr6	RLKGNK2	Tc06v2_g017380.1
CRIOLLO	BARq1BpPpchr6	RLKGNK2	Tc06v2_g017390.1
CRIOLLO	BARq1BpPpchr6	RLKGNK2	Tc06v2_g017400.1
CRIOLLO	BARq1BpPpchr6	RLKGNK2	Tc06v2_g017420.2
CRIOLLO	BARq1BpPpchr6	RLKGNK2	Tc06v2_g017430.1
CRIOLLO	BARq1BpPpchr6	RLKGNK2	Tc06v2_g017440.1
CRIOLLO	BARq1BpPpchr6	RLKGNK2	Tc06v2_g017470.1

CRIOLLO	BARq1BpPpchr6	RLP	Tc06v2_g016150.1
CRIOLLO	BARq1BpPpchr6	RLP	Tc06v2_g018760.1
CRIOLLO	BARq1BpPpchr6	T	Tc06v2_g015980.1
CRIOLLO	BARq1BpPpchr6	UNKNOWN	Tc06v2_g015100.1
CRIOLLO	BARq1BpPpchr6	UNKNOWN	Tc06v2_g016450.1
CRIOLLO	BARq1BpPpchr6	UNKNOWN	Tc06v2_g016480.1
CRIOLLO	BARq1BpPpchr6	UNKNOWN	Tc06v2_g016530.1
CRIOLLO	BARq1BpPpchr6	UNKNOWN	Tc06v2_g016540.1
CRIOLLO	BARq1BpPpchr6	UNKNOWN	Tc06v2_g017860.1
CRIOLLO	BARq1BpPpchr6	UNKNOWN	Tc06v2_g018570.1
CRIOLLO	BARq1BpPpchr6	UNKNOWN	Tc06v2_g018590.1
CRIOLLO	BARq1BpPpchr6	UNKNOWN	Tc06v2_g018600.1
CRIOLLO	BARq2BPPcchr2	UNKNOWN	Tc02v2_g013380.1
CRIOLLO	BARq2BPPcchr2	UNKNOWN	Tc02v2_g013390.1
CRIOLLO	BARq2BPPcchr2	UNKNOWN	Tc02v2_g013940.1
CRIOLLO	BARq2BPPcchr2	UNKNOWN	Tc02v2_g014020.1
CRIOLLO	BARq3BPPcchr3	CNL	Tc03v2_g026110.1*
CRIOLLO	BARq3BPPcchr3	CNL	Tc03v2_g026450.1*
CRIOLLO	BARq3BPPcchr3	N	Tc03v2_g026140.1*
CRIOLLO	BARq3BPPcchr3	N	Tc03v2_g026480.1*
CRIOLLO	BARq3BPPcchr3	NL	Tc03v2_g026160.1*
CRIOLLO	BARq3BPPcchr3	NL	Tc03v2_g026170.1*
CRIOLLO	BARq3BPPcchr3	NL	Tc03v2_g026190.1*
CRIOLLO	BARq3BPPcchr3	RLP	Tc03v2_g027150.1*
CRIOLLO	BARq3BPPcchr3	UNKNOWN	Tc03v2_g026130.1*
CRIOLLO	BARq3BPPcchr3	UNKNOWN	Tc03v2_g026470.1*
CRIOLLO	BARq3BPPcchr3	UNKNOWN	Tc03v2_g026890.1*
CRIOLLO	BARq4BPPcchr4	RLK	Tc04v2_g018750.1
CRIOLLO	BARq4BPPcchr4	RLP	Tc04v2_g019600.1
CRIOLLO	BARq4BPPcchr4	UNKNOWN	Tc04v2_g018480.1
CRIOLLO	BARq4BPPcchr4	UNKNOWN	Tc04v2_g018530.1
CRIOLLO	BARq4BPPcchr4	UNKNOWN	Tc04v2_g019390.1
CRIOLLO	BROPHY1chr4	RLKGNK2	Tc04v2_g001160.1
CRIOLLO	BROPHY1chr4	RLKGNK2	Tc04v2_g001170.1
CRIOLLO	BROPHY1chr4	RLP	Tc04v2_g000470.1
CRIOLLO	BROPHY2chr8	CNL	Tc08v2_g006690.1
CRIOLLO	BROPHY2chr8	CNL	Tc08v2_g006710.1
CRIOLLO	BROPHY2chr8	CNL	Tc08v2_g006730.1
CRIOLLO	BROPHY2chr8	MLO	Tc08v2_g005430.1
CRIOLLO	BROPHY2chr8	MLO	Tc08v2_g005450.1
CRIOLLO	BROPHY2chr8	RLP	Tc08v2_g006800.1
CRIOLLO	BROPHY2chr8	RLP	Tc08v2_g006810.1
CRIOLLO	BROPHY2chr8	RLP	Tc08v2_g007290.1
CRIOLLO	BROPHY2chr8	UNKNOWN	Tc08v2_g005750.1

CRIOLLO	BROPHY2chr8	UNKNOWN	Tc08v2_g006220.1
CRIOLLO	BROPHY3chr10	CN	Tc10v2_g011880.1
CRIOLLO	BROPHY3chr10	CNL	Tc10v2_g010220.1
CRIOLLO	BROPHY3chr10	CNL	Tc10v2_g010260.1
CRIOLLO	BROPHY3chr10	CNL	Tc10v2_g011850.1
CRIOLLO	BROPHY3chr10	CNL	Tc10v2_g011860.1
CRIOLLO	BROPHY3chr10	CNL	Tc10v2_g011910.1
CRIOLLO	BROPHY3chr10	CNL	Tc10v2_g011920.14
CRIOLLO	BROPHY3chr10	CNL	Tc10v2_g011930.1
CRIOLLO	BROPHY3chr10	CNL	Tc10v2_g012140.1
CRIOLLO	BROPHY3chr10	CNL	Tc10v2_g012150.1
CRIOLLO	BROPHY3chr10	CNL	Tc10v2_g012160.1
CRIOLLO	BROPHY3chr10	CNL	Tc10v2_g012180.2
CRIOLLO	BROPHY3chr10	CNL	Tc10v2_g013430.1
CRIOLLO	BROPHY3chr10	CNL	Tc10v2_g013440.1
CRIOLLO	BROPHY3chr10	N	Tc10v2_g011890.1
CRIOLLO	BROPHY3chr10	NL	Tc10v2_g012100.1
CRIOLLO	BROPHY3chr10	NL	Tc10v2_g012170.1
CRIOLLO	BROPHY3chr10	NL	Tc10v2_g013340.1
CRIOLLO	BROPHY3chr10	NL	Tc10v2_g013350.1
CRIOLLO	BROPHY3chr10	NL	Tc10v2_g013360.1
CRIOLLO	BROPHY3chr10	NL	Tc10v2_g013390.1
CRIOLLO	BROPHY3chr10	NL	Tc10v2_g013400.1
CRIOLLO	BROPHY3chr10	NL	Tc10v2_g013410.1
CRIOLLO	BROPHY3chr10	RLKGNK2	Tc10v2_g012610.1
CRIOLLO	BROPHY3chr10	RLP	Tc10v2_g010160.1
CRIOLLO	BROPHY3chr10	RLP	Tc10v2_g011560.1
CRIOLLO	BROPHY3chr10	RLP	Tc10v2_g011580.1
CRIOLLO	BROPHY3chr10	RLP	Tc10v2_g011640.1
CRIOLLO	BROPHY3chr10	UNKNOWN	Tc10v2_g010240.1
CRIOLLO	BROPHY3chr10	UNKNOWN	Tc10v2_g010310.1
CRIOLLO	BROPHY3chr10	UNKNOWN	Tc10v2_g011590.1
CRIOLLO	BROPHY3chr10	UNKNOWN	Tc10v2_g011600.1
CRIOLLO	BROPHY3chr10	UNKNOWN	Tc10v2_g011710.1
CRIOLLO	BROPHY3chr10	UNKNOWN	Tc10v2_g011900.1
CRIOLLO	BROPHY3chr10	UNKNOWN	Tc10v2_g012080.1
CRIOLLO	BROPHY3chr10	UNKNOWN	Tc10v2_g012700.1
CRIOLLO	BROPHY3chr10	UNKNOWN	Tc10v2_g012720.1
CRIOLLO	BROPHY3chr10	UNKNOWN	Tc10v2_g012750.1
CRIOLLO	BROPHY3chr10	UNKNOWN	Tc10v2_g012760.1
CRIOLLO	BROPHY3chr10	UNKNOWN	Tc10v2_g012770.2
CRIOLLO	BROPHY3chr10	UNKNOWN	Tc10v2_g012830.1
CRIOLLO	BROPHY3chr10	UNKNOWN	Tc10v2_g012850.1
CRIOLLO	BROPHY3chr10	UNKNOWN	Tc10v2_g012930.1

CRIOLLO	BROPHY3chr10	UNKNOWN	Tc10v2_g012980.1
CRIOLLO	BROPHY3chr10	UNKNOWN	Tc10v2_g013370.1
CRIOLLO	BROPHY3chr10	UNKNOWN	Tc10v2_g013380.1
CRIOLLO	BROPHY3chr10	UNKNOWN	Tc10v2_g013420.1
MATINA	AKAfolICS100chr1	UNKNOWN	3211130
MATINA	AKAfolICS100chr1	UNKNOWN	3211656
MATINA	AKAfolICS100chr1	UNKNOWN	3212639
MATINA	AKAfolICS100chr1	UNKNOWN	3212940
MATINA	AKAfolICS100chr1	UNKNOWN	3214102
MATINA	AKAfolICS100chr10	UNKNOWN	3244038
MATINA	AKAfolICS100chr3	CN	3344436
MATINA	AKAfolICS100chr3	CNL	3344063
MATINA	AKAfolICS100chr3	CNL	3344449
MATINA	AKAfolICS100chr3	CNL	3344500
MATINA	AKAfolICS100chr3	N	3344016
MATINA	AKAfolICS100chr3	N	3344488
MATINA	AKAfolICS100chr3	NL	3344454
MATINA	AKAfolICS100chr3	RLP	3343189
MATINA	AKAfolICS100chr3	UNKNOWN	3343513
MATINA	AKAfolICS100chr3	UNKNOWN	3344057
MATINA	AKAfolICS100chr3	UNKNOWN	3344431
MATINA	AKAprrHchr1	CNL	3184683
MATINA	AKAprrHchr1	CNL	3184894
MATINA	AKAprrHchr1	RLP	3184106
MATINA	AKAprrHchr1	RPW8NL	3196185
MATINA	AKAprrHchr1	RPW8NL	3196210
MATINA	AKAprrHchr1	UNKNOWN	3184356
MATINA	AKAprrHchr1	UNKNOWN	3184361
MATINA	AKAprrHchr1	UNKNOWN	3185860
MATINA	AKAprrHchr8	RLP	3506200
MATINA	AKAprrHchr8	RLP	3507182
MATINA	AKAprrHchr8	RLP	3508342
MATINA	AKAprrHchr8	UNKNOWN	3507278
MATINA	AKAprrICS100chr6_1	UNKNOWN	3464167
MATINA	AKAprrICS100chr6_1	UNKNOWN	3464843
MATINA	AKAprrICS100chr6_1	UNKNOWN	3464909
MATINA	AKAprrICS100chr6_1	UNKNOWN	3465023
MATINA	AKAprrICS100chr6_2	CNL	3446023
MATINA	AKAprrICS100chr6_2	CNL	3446028
MATINA	AKAprrICS100chr6_2	RLP	3445147
MATINA	AKAprrICS100chr6_2	RLP	3446117
MATINA	AKAprrICS95chr2	CNL	3267585
MATINA	AKAprrICS95chr2	NL	3267613
MATINA	AKAprrICS95chr2	NL	3267643

MATINA	AKAprrICS95chr2	UNKNOWN	3267416
MATINA	AKAprrICS95chr2	UNKNOWN	3267579
MATINA	AKAprrICS95chr2	UNKNOWN	3267589
MATINA	AKAprrICS95chr2	UNKNOWN	3267623
MATINA	AKAprrICS95chr2	UNKNOWN	3267653
MATINA	AKAprrICS95chr2	UNKNOWN	3268296
MATINA	AKAprrICS95chr2	UNKNOWN	3268309
MATINA	BARq1BPPcchr1	CNL	3222320
MATINA	BARq1BPPcchr1	CNL	3222330
MATINA	BARq1BPPcchr1	CNL	3222462
MATINA	BARq1BPPcchr1	CNL	3222500
MATINA	BARq1BPPcchr1	RLP	3223656
MATINA	BARq1BPPcchr1	RLP	3227564
MATINA	BARq1BPPcchr1	UNKNOWN	3222923
MATINA	BARq1BPPcchr1	UNKNOWN	3223367
MATINA	BARq1BPPcchr1	UNKNOWN	3224250
MATINA	BARq1BPPctchr6	CNL	3462782
MATINA	BARq1BPPctchr6	CNL	3462815
MATINA	BARq1BPPctchr6	RLP	3462662
MATINA	BARq1BPPctchr6	RLP	3462976
MATINA	BARq1BPPctchr6	UNKNOWN	3462709
MATINA	BARq1BpPpchr6	RLK	3451646
MATINA	BARq1BpPpchr6	RLK	3451662
MATINA	BARq1BpPpchr6	RLK	3451708
MATINA	BARq1BpPpchr6	RLK	3451759
MATINA	BARq1BpPpchr6	RLK	3451845
MATINA	BARq1BpPpchr6	RLK	3451861
MATINA	BARq1BpPpchr6	RLKGNK2	3450801
MATINA	BARq1BpPpchr6	RLKGNK2	3450875
MATINA	BARq1BpPpchr6	RLKGNK2	3450891
MATINA	BARq1BpPpchr6	RLKGNK2	3450973
MATINA	BARq1BpPpchr6	RLKGNK2	3450996
MATINA	BARq1BpPpchr6	RLKGNK2	3451011
MATINA	BARq1BpPpchr6	RLKGNK2	3451045
MATINA	BARq1BpPpchr6	RLKGNK2	3451072
MATINA	BARq1BpPpchr6	RLKGNK2	3451094
MATINA	BARq1BpPpchr6	RLKGNK2	3451139
MATINA	BARq1BpPpchr6	RLKGNK2	3451166
MATINA	BARq1BpPpchr6	RLKGNK2	3451197
MATINA	BARq1BpPpchr6	RLKGNK2	3451228
MATINA	BARq1BpPpchr6	RLKGNK2	3451244
MATINA	BARq1BpPpchr6	RLKGNK2	3451271
MATINA	BARq1BpPpchr6	RLKGNK2	3451315
MATINA	BARq1BpPpchr6	RLP	3449263

MATINA	BARq1BpPpchr6	RLP	3452732
MATINA	BARq1BpPpchr6	T	3452900
MATINA	BARq1BpPpchr6	UNKNOWN	3449561
MATINA	BARq1BpPpchr6	UNKNOWN	3449566
MATINA	BARq1BpPpchr6	UNKNOWN	3449575
MATINA	BARq1BpPpchr6	UNKNOWN	3450344
MATINA	BARq1BpPpchr6	UNKNOWN	3452116
MATINA	BARq1BpPpchr6	UNKNOWN	3452124
MATINA	BARq1BpPpchr6	UNKNOWN	3452184
MATINA	BARq1BpPpchr6	UNKNOWN	3452246
MATINA	BARq1BpPpchr6	UNKNOWN	3453912
MATINA	BARq2BPPcchr2	RLKGNK2	3318311
MATINA	BARq2BPPcchr2	UNKNOWN	3317751
MATINA	BARq2BPPcchr2	UNKNOWN	3317758
MATINA	BARq2BPPcchr2	UNKNOWN	3318761
MATINA	BARq2BPPcchr2	UNKNOWN	3318922
MATINA	BARq3BPPcchr3	CN	3344436*
MATINA	BARq3BPPcchr3	CNL	3344063*
MATINA	BARq3BPPcchr3	CNL	3344449*
MATINA	BARq3BPPcchr3	CNL	3344500*
MATINA	BARq3BPPcchr3	N	3344016*
MATINA	BARq3BPPcchr3	N	3344488*
MATINA	BARq3BPPcchr3	NL	3344454*
MATINA	BARq3BPPcchr3	RLP	3343189*
MATINA	BARq3BPPcchr3	UNKNOWN	3343513*
MATINA	BARq3BPPcchr3	UNKNOWN	3344057*
MATINA	BARq3BPPcchr3	UNKNOWN	3344431*
MATINA	BARq4BPPcchr4	RLK	3379026
MATINA	BARq4BPPcchr4	RLP	3375376
MATINA	BARq4BPPcchr4	UNKNOWN	3378443
MATINA	BARq4BPPcchr4	UNKNOWN	3379338
MATINA	BARq4BPPcchr4	UNKNOWN	3379407
MATINA	BROPHY1chr4	RLP	3364140
MATINA	BROPHY1chr4	RLKGNK2	3365094
MATINA	BROPHY2chr8	CNL	3502049
MATINA	BROPHY2chr8	CNL	3502107
MATINA	BROPHY2chr8	CNL	3502117
MATINA	BROPHY2chr8	MLO	3504014
MATINA	BROPHY2chr8	MLO	3504085
MATINA	BROPHY2chr8	RLP	3501305
MATINA	BROPHY2chr8	RLP	3501983
MATINA	BROPHY2chr8	RLP	3501992
MATINA	BROPHY2chr8	UNKNOWN	3503001
MATINA	BROPHY2chr8	UNKNOWN	3503681

MATINA	BROPHY3chr10	CNL	3254341
MATINA	BROPHY3chr10	CNL	3256755
MATINA	BROPHY3chr10	CNL	3257002
MATINA	BROPHY3chr10	CNL	3257102
MATINA	BROPHY3chr10	CNL	3257324
MATINA	BROPHY3chr10	CNL	3257344
MATINA	BROPHY3chr10	CNL	3257371
MATINA	BROPHY3chr10	CNL	3258828
MATINA	BROPHY3chr10	CNL	3258892
MATINA	BROPHY3chr10	NL	3253595
MATINA	BROPHY3chr10	NL	3257309
MATINA	BROPHY3chr10	NL	3258821
MATINA	BROPHY3chr10	NL	3258850
MATINA	BROPHY3chr10	RLKGNK2	3258102
MATINA	BROPHY3chr10	RLP	3254087
MATINA	BROPHY3chr10	RLP	3256681
MATINA	BROPHY3chr10	RLP	3256715
MATINA	BROPHY3chr10	RLP	3256785
MATINA	BROPHY3chr10	UNKNOWN	3253920
MATINA	BROPHY3chr10	UNKNOWN	3254276
MATINA	BROPHY3chr10	UNKNOWN	3254281
MATINA	BROPHY3chr10	UNKNOWN	3254467
MATINA	BROPHY3chr10	UNKNOWN	3255072
MATINA	BROPHY3chr10	UNKNOWN	3255812
MATINA	BROPHY3chr10	UNKNOWN	3256700
MATINA	BROPHY3chr10	UNKNOWN	3256704
MATINA	BROPHY3chr10	UNKNOWN	3256739
MATINA	BROPHY3chr10	UNKNOWN	3256837
MATINA	BROPHY3chr10	UNKNOWN	3256909
MATINA	BROPHY3chr10	UNKNOWN	3256939
MATINA	BROPHY3chr10	UNKNOWN	3256961
MATINA	BROPHY3chr10	UNKNOWN	3257050
MATINA	BROPHY3chr10	UNKNOWN	3257298
MATINA	BROPHY3chr10	UNKNOWN	3257945
MATINA	BROPHY3chr10	UNKNOWN	3258221
MATINA	BROPHY3chr10	UNKNOWN	3258264
MATINA	BROPHY3chr10	UNKNOWN	3258360
MATINA	BROPHY3chr10	UNKNOWN	3258374
MATINA	BROPHY3chr10	UNKNOWN	3258498
MATINA	BROPHY3chr10	UNKNOWN	3259166
MATINA	BROPHY3chr10	UNKNOWN	3259285
MATINA	BROPHY3chr10	UNKNOWN	3259317
MATINA	BROPHY3chr10	UNKNOWN	3259346
